# Whole body periodic acceleration (pGz) improves endotoxin induced cardiomyocyte contractile dysfunction and attenuates the inflammatory response in mice

**DOI:** 10.1016/j.heliyon.2021.e06444

**Published:** 2021-03-12

**Authors:** Jose A. Adams, Jose R. Lopez, Arkady Uryash, Marvin A. Sackner

**Affiliations:** a Division of Neonatology, Mount Sinai Medical Center, Miami Beach, FL, USA; b Department of Research, Mount Sinai Medical Center, Miami Beach, FL, USA; c Department of Medicine, Mount Sinai Medical Center, Miami Beach, FL, USA

**Keywords:** Sepsis, LPS, Whole body periodic acceleration, Calcium, Nitric oxide, Endothelial nitric oxide, pGz, Lipopolysaccharide, Cytokines, Cardiomyocytes, Sepsis induced cardiomyopathy

## Abstract

Sepsis-induces myocardial contractile dysfunction. We previously showed that whole body periodic acceleration (pGz), the sinusoidal motion of the supine body head-foot ward direction significantly improves survival and decreases microvascular permeability in a lethal model of sepsis. We tested the hypothesis that pGz improves LPS induced cardiomyocyte contractile dysfunction and decreases LPS pro-inflammatory cytokine response when applied pre- or post-treatment. Isolated cardiomyocytes were obtained from mice that received LPS who had been pre-treated with pGz for three days (pGz-LPS) or control. Peak shortening (PS), maximal velocity of shortening (+dL/dt), and relengthening (-dL/dt) as well as diastolic intracellular calcium concentration ([Ca^+2^]_d_), sodium ([Na^+^]_d_), reactive oxygen species (ROS), and cardiac troponin (cTnT) production were measured. LPS decreased PS, +dL/dt, and -dL/dt, by 37%, 41% and 35% change respectively (p < 0.01), increased [Ca^+2^]_d_, [Na^+^]_d_, ROS, and cTnT by 343%, 122%, 298%, and 610% change respectively (p < 0.01) compared to control. pGz pre-treatment attenuated the parameters mentioned above. In a separate cohort, the effects of a lethal dose of LPS on protein expression of nitric oxide synthases (iNOS, eNOS, nNOS), pro- and anti-inflammatory cytokines in hearts of mice was studied in pre-treated with pGz for three days prior to LPS (pGz-LPS) and post-treated with pGz 30 min after LPS (LPS-pGz) were determined. LPS increased expression of early and late iNOS and decreased expression of eNOS, phosphorylated eNOS (p-eNOS), and nNOS. Both pre- and post-treatment with pGz markedly reduced early and late pro-inflammatory surge. Therefore, pre- and post-treatment with pGz improves LPS-induced cardiomyocyte dysfunction, decreases iNOS expression, and increases cytoprotective eNOS and nNOS, with decreased pro-inflammatory response. Such results have potential for translation to benefit outcomes in human sepsis.

## 1 Introduction

Sepsis affects more than 1.5 million adults in the USA, with mortality rates of 15–30% (Hershey and Kahn, 2017). Cardiac manifestations are a well-recognized complication of sepsis and denoted as sepsis-induced cardiomyopathy (SIC) with a 10–70% prevalence. This complication is manifested as contractile dysfunction, decreased systolic contractility with compensatory diastolic ventricular dilatation, and severe decrease of diastolic compliance (Walley, 2018). Other factors associated with SIC include increased pathogen-associated toll-like receptors TLR4, increased pro-inflammatory cytokine levels, elevated iNOS derived nitric oxide (NO), reactive oxygen species (ROS), and dysfunctional calcium handling (Martin et al., 2019). Homeostasis of intracellular calcium is vital to normal myocardial contraction and relaxation, and alterations in calcium homeostasis in cardiomyocytes have been shown to induce both functional and structural changes in SIC (Celes et al., 2013).

Escherichia coli (E. coli) endotoxin processed as lipopolysaccharide (LPS) produces a dose-dependent, systemic inflammatory response and serves as a model for the upregulation of the inflammatory cascade. The inflammatory response is accompanied by large (micromolar) quantities of nitric oxide from inducible nitric oxide synthase (iNOS), which in large part is responsible for cardiac dysfunction and adverse hemodynamic changes in sepsis. Small quantities of nitric oxide (nanomolar) released from the endothelium via endothelial nitric oxide (eNOS) by shear stress is anti-inflammatory and cardioprotective (Daiber et al., 2019; Predescu et al., 2005). Increased shear stress can be produced by applying Whole Body Periodic Acceleration (pGz) by means of a motorized platform that adds pulses to the circulation, which activates eNOS with subsequent release of NO into the circulation (Adams et al., 2003). We previously demonstrated that pGz increases eNOS activity through the phosphoinositide 3-kinase protein kinase B signaling pathway (PI3K-AKT) and reduces intracellular calcium overload in endothelial cells, cardiomyocytes from mice, rats, pigs, and humans (Lopez et al., 2017, 2018; Wu et al., 2009, 2012). Further, both pre- and post-treatment with pGz significantly increased survival and decreased microvascular permeability in a lethal LPS mouse model (Adams et al., 2019).

In the current study, we tested the hypothesis that pGz improves LPS induced cardiomyocyte contractile dysfunction and decreases LPS pro-inflammatory cytokine response when applied as a pre- or post-treatment strategy.

## 2 Materials and methods

### 2.1 Animal preparation

The study protocol conforms to the Guide for the Care and Use of Laboratory Animals published by the National Institutes of Health (NIH Publication No. 85–23, revised 1996). The Institutional Animal Care and Use Committee of Mount Sinai Medical Center (accredited by AAALAC - Association for Assessment and Accreditation of Laboratory Animal Care International) and Office for Laboratory Animal Welfare (Assurance # A3044-01) approved this protocol No. 17-20-A-04. Mice studied were C57BL/6J (The Jackson Laboratory, Bar Harbor, ME) 3-month old mice of either sex weight range 20–25 gm. Animals were randomized to each of the studies via computer-generated codes.

### 2.2 Protocols

Two studies were carried out: (1) *LPS and pGz Cellular Data*. We tested the hypothesis that pGz pre-treatment reduces contractile dysfunction as detected in isolated cardiomyocytes. *Wt* 3 month old mice of either sex (N = 25, non-anesthetized nor sedated) were randomized to five groups of 5 animals each: ***a)*** Control; ***b)*** Control treated with three days of pGz (pGz); ***c)*** Lipopolysaccharide (LPS) mice that received an intraperitoneal injection of *E.coli/LPS* (Sigma Aldrich, St. Luis, MO, USA) to induce endotoxemia at a dose of 10 mg/kg, a dose demonstrated to ensure cardiac dysfunction without mortality (Poon et al., 2003); ***d)*** pre-treated with pGz for three days followed by injection of LPS (pGz-LPS), ***e)*** pre-treated with L-NAME (L-NG-Nitroarginine methyl ester) for seven days then with pGz for three days followed by administration of LPS (L-NAME-pGz-LPS). In this last group, we tested whether the effects of pGz were related to endothelial release of nitric oxide. L-NAME, (1.5 mg/ml) was given in their drinking water for seven days. L-NAME's dose was based on preliminary experiments, which revealed that mice drank approximately 2 ml/day; this represents an oral dose of approximately 100 mg/kg of body weight per day. In all groups, the mice were euthanized upon completion of their respective protocols for cardiomyocytes contractile measurements, [Ca^2+^]_d_, [Na^+^]_d_, ROS production, and plasma cTnT concentration.

In the second study, (2) *Protein Expression and Cytokines*, we tested the effects of pGz on NOS isoforms and cytokine protein expression in mice hearts with SIC. Sixty four mice (C57BL/6J) of 3 months of age of either sex were randomly assigned (computer-generated code) to one of four groups prior to LPS inoculation; ***a)*** Pre-treatment with pGz (pGz-LPS, N = 16) 1 h per day for three days; ***b)*** Post-treatment with pGz (LPS-pGz, N = 16) starting immediately after LPS and continued for 1 h; ***c)*** LPS (LPS, N = 16), these mice received an intraperitoneal injection of *E.coli/LPS*, at a lethal dose of 40 mg/kg diluted in phosphate buffered saline, total volume 0.1ml, a model in which 100% mortality occurs within 1440 min (Adams et al., 2019), ***d)***; Control (CONT) (N = 16) in which the same volume of phosphate buffer was given i.p. In this second study, 8 out of the 16 mice were sacrificed at 90 min and the other half at 360 min after LPS administration or buffer. The time periods were chosen on the basis of our previous work, where at both 90 and 360 min all animals survived (Adams et al., 2019). A schematic of the above protocols can be found in Supplementary File.

### 2.3 Platform specifications

pGz is imparted using a motion platform. In mice the reciprocal platform set at a frequency of 480 cycles/minute (8 Hz) and acceleration in the z-plane (Gz) of ± 3.0 m/sec^2^. This platform has been previously described (Lopez et al., 2016; Uryash et al., 2015a) and schematics and descriptions could be found in Supporting Information File (Rauch et al., 2010).

### 2.4 LPS and pGz Cellular Data

#### 2.4.1 Cardiomyocyte isolation

Prior to anesthesia with ketamine (100 mg/kg) and xylazine (5 mg/kg), mice were heparinized (1000 U/kg; i.p.), and hearts were rapidly removed and mounted onto a temperature-controlled (37 °C) perfusion system. Single ventricular cardiomyocytes were isolated according to a previously established protocol (Liao and Jain, 2007). All cardiomyocytes used in this study (within six hour after isolation) were rod-shaped, had well-defined striations spacing, and when perfused with normal Tyrode solution (2 mM Ca^2+^) did not spontaneously contract.

#### 2.4.2 Determinations of [Ca^2+^]_d_ and [Na^+^]_d_

For determination of [Ca^2+^]_d_ and [Na^+^]_d_, isolated cardiomyocytes were impaled with the Ca^2+^ or Na^+^ double-barreled selective microelectrodes, and the potentials from membrane potential (Vm) and VCa_E_ or VNa_E_ were recorded via high impedance amplifier (WPI SYS-773, Sarasota, FL, USA) (Eltit et al., 2013). The potential from the Vm barrel (3 M KCl) (resting membrane potential (Vm)) was subtracted electronically from VCa_E_ or VNa_E_, to produce a differential Ca^2+^-specific potential (V_Ca_) or Na^+^-specific potential (V_Na_) that represents the [Ca^2+^]_d_ and [Na^+^]_d_ respectively. The potentials were recorded using a high-impedance amplifier >1011 MΩ (FD-223; WPI, Sarasota, FL) Recorded potentials were filtered with a low-pass filter (30 kHz) acquired using AxoGraph software (version 4.6; Axon Instruments, Foster City, CA) sampling ata frequency of 1,000 Hz. The signals were stored for further analysis. A low pass filter (LPF30, WPI, Sarasota, FL) was used to smooth the signal. LPF are filters used to pass signals with a frequency lower than a selected cutoff frequency and attenuates signals with frequencies higher than the cutoff frequency LPF do not modify or interfere with the time course of the recorded potentials because of the slow response of the ion-selective microelectrodes. No attempts have been made to quantify the intracellular Ca^2+^ concentration-time course, due to the known ion-selective microelectrodes' response time limitation.

#### 2.4.3 Cell shortening

A video-based edge-detection system (IonOptix, IonOptix Corporation, Milton, Massachusetts) which allows tracking cardiomyocytes edges changes during contraction and relaxation (shortening-lengthening) was used. Isolated cardiomyocytes were allowed to adhere to a glass coverslip in a Warner chamber mounted on the stage of an inverted microscope. Myocytes were perfused with Tyrode's solution. Field stimulation of the cardiomyocytes was performed with a pair of platinum electrodes at a frequency of 1 Hz (2 ms pulse duration ~1.5x threshold voltage). The edges of the cardiomyocytes were continuously tracked during contraction and relaxation using a SoftEdge MyoCam system (IonOptix Corporation, Milton, Massachusetts) (IonOptix, Milton, Massachusetts). The following parameters were measured: i) diastolic sarcomere length which was determined after a 30-s stimulation (2 ms pulse duration ~1.5x threshold voltage) in quiescent cardiomyocytes; ii) peak shortening (PS), indicative of peak ventricular contractility; iii) maximal velocity of shortening (+dL/dt), indicative of ventricular pressure rise; iv) maximal velocity of lengthening (−dL/dt), indicative of ventricular pressure fall. Data was collected from only rod-shaped cardiomyocytes with good striation, a diastolic sarcomere length >1.8 μm, and no spontaneous contraction in the presence of normal extracellular Ca^2+^ concentration. Experiments were conducted at 37 °C.

#### 2.4.4 Reactive oxygen species and cardiac troponin T

Cardiomyocyte ROS production was measured with the ROS-sensitive fluorescent probe 5-(-6)-chloromethyl-2′,7′dichlorodihydrofluorescein diacetate (CM-H2DCFDA) (Sigma-Aldrich, St. Louis, MO, USA), and plasma levels of cardiac troponin T (cTnT) quantified by ELISA (Boehringer Mannheim, Indianapolis, IN, USA). Blood samples for cTnT determination were drawn at the conclusion of the treatment protocol from the mice's tail vein, as previously described (Lopez et al., 2017).

### 2.5 Cytokine and protein expressions

#### 2.5.1 Protein and cytokine analysis

Hearts from the high dose LPS were harvested at 90 and 360 min after LPS infusion and processed for protein analysis using western blot techniques previously reported (Adams et al., 2019; Uryash, Bassuk, 2015a). These periods were chosen in order to have two specific periods with adequate survival of animals in both treated and control groups based on survival data (Adams et al., 2019). Briefly, homogenized mouse hearts were processed using a one-step protein extraction kit (Millipore Corporation, Billerica, MA). Then total protein concentrations were measured by the BCA Protein Assay (Thermo Fisher Scientific, Waltham, MA, USA) on a SpectraMax Plate Reader (Molecular Devices, Sunnyvale, CA, USA). Individual proteins of interest were then analyzed by western blot. Equal amounts of total protein were separated on 4–12% NuPAGE Novex Bis-Tris SDS-PAGE Gels (Life Technologies, Carlsbad, CA, USA) and transferred to nitrocellulose membrane (Bio-Rad, Hercules, CA, USA). The transfer membrane was treated with a blocking agent (GE Bio-Sciences, Piscataway, NJ, USA) and probed with primary, fluorescein-linked secondary antibodies as well as anti-fluorescein alkaline phosphatase conjugate.

The following primary antibodies were used: eNOS (5589, 1:2000), p-eNOS (184154, 1:2000 Ser 1177), nNOS (5586, 1:2000), iNOS (15323, 1:2000), TNFα (6671, 1:5000), IL-1β (9787, 1:2000), IL-6 (9324, 1:2000), IL-10 (9969, 1:2000), NFkB-p65 (32536, 1:1000), GAPDH (9545, 1:10000) (Abcam, Cambridge, MA, USA). Protein signals were visualized using Enhanced Chemifluorescence kit (ECF) and Storm 860 Imaging System (GE Bio-Sciences, Piscataway, NJ, USA). The Storm 860 Imaging System exhibits a linear response to fluorescent signal intensities, and protein levels were quantified using MYImage Analysis software (Thermo Fisher Scientific, Waltham, MA, USA). Optical units were first standardized to GAPDH protein loading control and then referenced to control levels of individual proteins (Supporting Information File).

### 2.6 Solutions

Cardiomyocytes were perfused with Tyrode solution aerated with 95% O_2_ and 5% CO_2_. The composition (in mM) of the Tyrode solution is; NaCl 130, KCl 2.68, CaCl_2_ 1.8, MgCl_2_ 1, NaHCO_3_ 12, NaH_2_PO_4_ 0.4, glucose 5, and pH 7.4. All experiments were performed at 37 °C.

### 2.7 Euthanasia

Humane endpoints for euthanasia was carried out in all animals for the initial 24 h after LPS injection. Behavioral Scoring criteria described by Shrum et al. (2014) was amended to include stool quality as additional criteria, with a maximum score of 32 (Supporting Information File) Scoring was performed every 30 min after LPS for the first 2 h, thereafter every 1hr for until 1440 min Euthanasia was performed within 15 min of reaching a score of 28. by a dose of Ketamine 90 mg/kg and Xylazine 25 mg/kg, followed by pentobarbital 100 mg/kg IP, until the absence of corneal and pedal reflex, and no electrical activity on ECG, and decapitation via guillotine, a method approved by the American Veterinary Medical Association Guidelines on Euthanasia (2013).

### 2.8 Statistical analyses

Continuous variables were evaluated by analysis of variance (ANOVA) for repeated measures. For variables with significant differences, post hoc analysis was done using Tukey HSD for equal or unequal sample size. Statistical analyses were performed using STATISTICA (StatSoft Inc., Tulsa, OK) and GraphPad Prism 9 (GraphPad Software, Inc., San Diego, CA). Changes from one condition (x) to another (y) were calculated and reported as (y-x)/x ∗100 (% change from x). The sample size was calculated using STATISTICA based on power analysis with α = 0.05 and power 0.80. A p value of < 0.05 was considered statistically significant. Supporting Information File contains power calculation used to arrive at sample size. Values are expressed as means ± SD, with “n” representing the number of cardiomyocytes in which measurements were carried out and “N” representing the number of mice used to isolate the cardiomyocytes.

## 3 Results

### 3.1 pGz reversed the LPS induced cardiomyocyte contractile dysfunction

Impaired cardiac function is usually the most predominant clinical presentation in septic patients manifested as decreased ejection fraction and myocardial contractility (Niederbichler et al., 2006).

**Figure 1:**
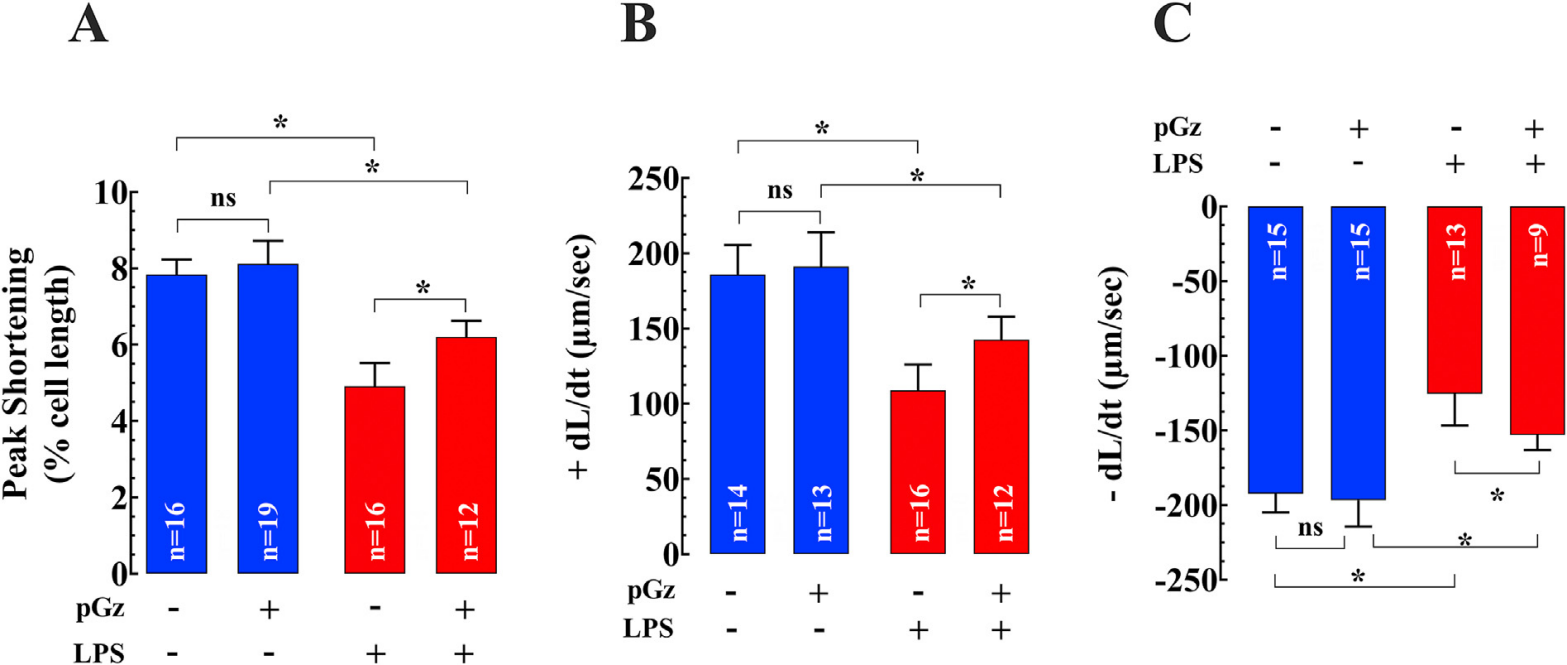
Effects of pGz on LPS induced cardiomyocyte contractile dysfunction. The effects of pGz on the LPS-induced cardiomyocyte contractile dysfunction were evaluated on isolated quiescent cardiomyocyte. LPS significantly reduced (A) Peak shortening (% of cell length) (n = 16–19, N = 5, p < 0.05); (B) Maximal velocity of shortening (+dL/dt) (n = 13–14, N = 5, p < 0.05); (C) Maximal velocity of re-lengthening (-dL/dt) (n = 15, N = 5, p < 0.05). Pretreatment with pGz significantly improved peak shortening (n = 16–19, N = 5, p < 0.05), +dL/dt (n = 13–16, N = 5, p < 0.05), and -dL/dt (n = 13–15, N = 5, p < 0.05) (A, B, and C) compared to untreated cardiomyocytes. Data are presented as means ± SD (∗p < 0.05).

We studied the effects of LPS on contractile properties in isolated cardiomyocytes and whether pGz could reverse these changes. LPS reduced peak shortening (PS) by 37% (n = 16–19, N = 5, *p* < 0.05), the maximal velocity of shortening (+dL/dt) by 41%- (n = 13–14, N = 5, *p* < 0.05), and relengthening (-dL/dt) by 35% (n = 15, N = 5, *p* < 0.05) compared to non-LPS treated cardiomyocytes (Figures 1A, B and C). pGz pre-treatment improved PS by 26% (n = 16–19, N = 5, *p* < 0.05), the +dL/dt by 31% (n = 13–16, N = 5, *p* < 0.05), and -dL/dt by 22% change- (n = 13–15, N = 5, *p* < 0.05) compared to LPS treated cells but pGz untreated (Figures 1A, B, and C).

### 3.2 Effects of pGz on LPS induced elevation of [Ca^2+^]_d,_ [ Na^+^]_d_, ROS, and cTnT in cardiomyocytes

**Figure 2:**
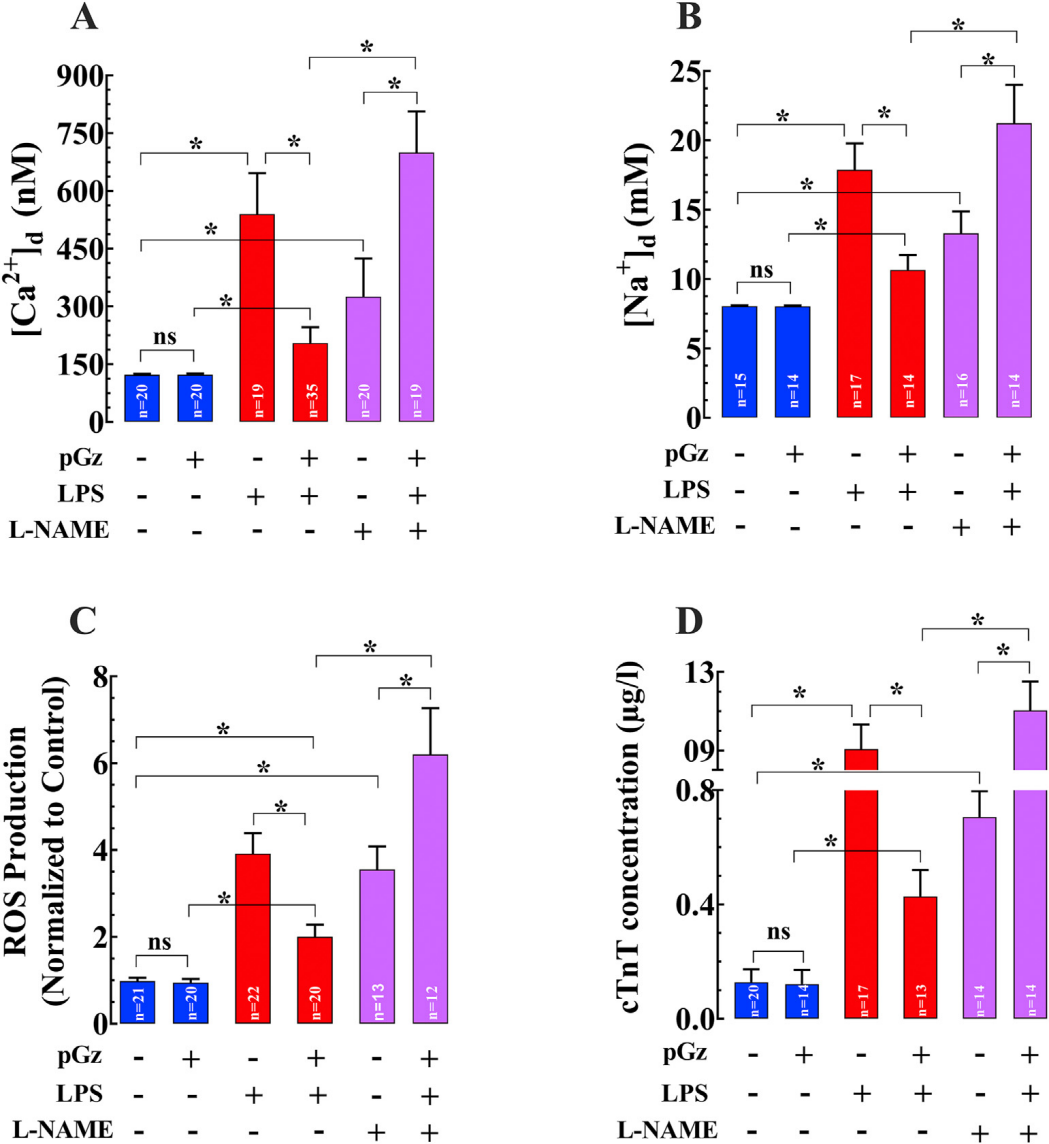
Beneficial effects of pGz on LPS mediated elevation of [Ca^2+^]_d_, [Na^+^]_d_, ROS and cTnT. LPS induced increase in (A) [Ca^2+^]_d_ (n = 17–20, N = 5, p < 0.05), (B) [Na^+^]_d_ (n = 15–17, N = 5, p < 0.05), (C) ROS (n = 20, N = 5, p < 0.05), and (D) cTnT (n = 17–20, N = 5, p < 0.05) compared to untreated cardiomyocytes. pGz pretreatment induced a cardioprotection by a significant reduction in (A) [Ca^2+^]_d_ (n = 17–13, N = 5, p < 0.05), (B) [Na^+^]_d_ (n = 14–17, N = 5, p < 0.05), (C) ROS (n = 20, N = 5, p < 0.05), and (D) cTnT (n = 13–17, N = 5, p < 0.05). L-NAME abolished the cardioprotection induced by pGz on LPS treated cardiomyocytes (n = 14–31, N = 5, p < 0.05) (Figures 2A-D). Data are presented as means ± SD (∗p < 0.05).

The effects of LPS on [Ca^2+^]_d_, [Na^+^]_d_, ROS production were evaluated in quiescent cardiomyocytes and cardiac troponin T concentration (cTcT) in blood. In isolated cardiomyocytes, LPS produced a significant increase in [Ca^2+^]_d_ by 343% (n = 17–20, N = 5, *p* < 0.05) (Figure 2A), [Na^+^]_d_ by 122% (n = 15–17, N = 5, *p* < 0.05) (Figure 2B), ROS generation by 298% (n = 20, N = 5, *p* < 0.05) (Figure 2C), and cTnT by 610% (n = 17–20, N = 5, *p* < 0.05) (Figure 2D) change compared to untreated cardiomyocytes. pGz pretreatment induced significant cardioprotection by reducing [Ca^2+^]_d_ 62% (n = 17–13, N = 5, *p* < 0.05), [Na^+^]_d_ 40% (n = 14–17, N = 5, *p* < 0.05), ROS 49% (n = 20, N = 5, *p* < 0.05), and cTnT 53% (n = 13–17, N = 5, *p* < 0.05) compared to LPS exposed but pGz untreated cardiomyocytes. In addition, we found that the non-selective nitric oxide inhibitor (L-NAME) abolished the beneficial effects of pGz on [Ca^2+^]_d,_ [Na^+^]_d_, ROS, and cTnT (n = 14–31, N = 5, *p* < 0.05) (Figures 2A, B, C, and D).

### 3.3 Protein and cytokine analysis

**Figure 3:**
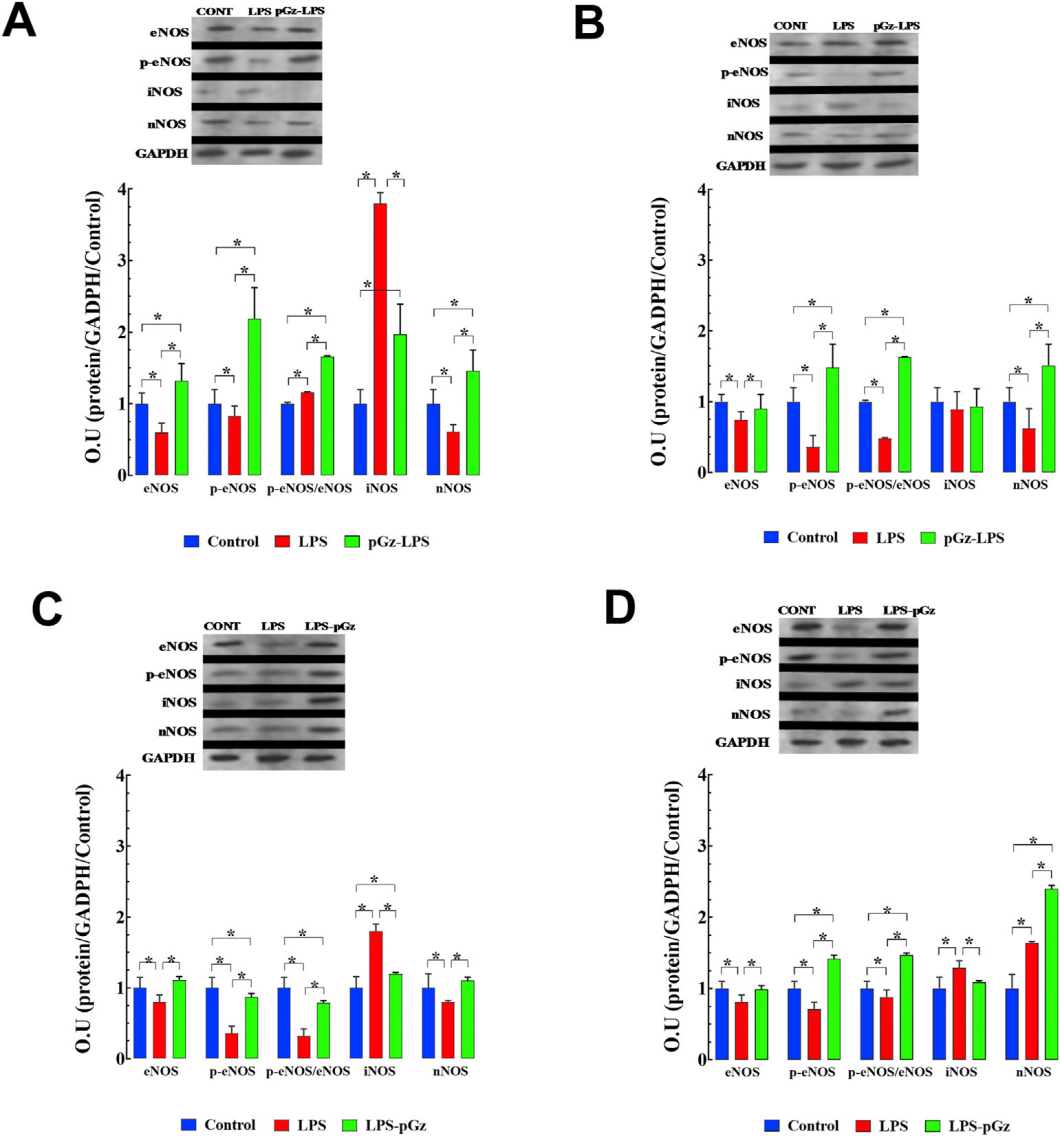
pGz reversed the LPS effects on expression of nitric oxide synthases in mice heart. LPS produced a significant decrease in eNOS, p-eNOS, and nNOS at 90 and 360 min and significantly increased iNOS at 90 min. (A) pGz mice pre-treatment (pGz-LPS) increased expression of eNOS, p-eNOS, nNOS, and decreased iNOS compared to LPS-treated mice at 90 min and 360 min (B) pGz mice post-treatment (LPS-pGz) also increased eNOS, p-eNOS, nNOS and decreased iNOS compared to LPS treated mice, at 90 min (C) and 360min (D). Supporting Information File contains uncropped Western Blots. Data are expressed as mean ± SD (∗p < 0.05).

Nitric oxide protein isoforms expression was evaluated in the most severe LPS endotoxemia model, early (90min) and late (360 min) after LPS in pGz pre- and post-treated mice. These periods were chosen based on of survival data from our previous study (Adams et al., 2019). At 90 min after LPS, there was a decrease of eNOS, p-eNOS as well as nNOS, with a 280% increase in iNOS compared to control. pGz-LPS increased eNOS, p-eNOS, and nNOS 120%, 164%, and 140%, change respectively from LPS values. pGz-LPS markedly decreased iNOS upregulation by 48% change from LPS values. At 360 min after LPS, there were decreases of eNOS, p-eNOS, and nNOS of 74%, 36%, 62% change respectively compared to control, without significant change of iNOS expression induced by LPS. pGz-LPS also increased eNOS, p-eNOS, and nNOS by 22%, 310%, and 144% change compared to LPS respectively (Figure 3 A, B).

**Figure 4:**
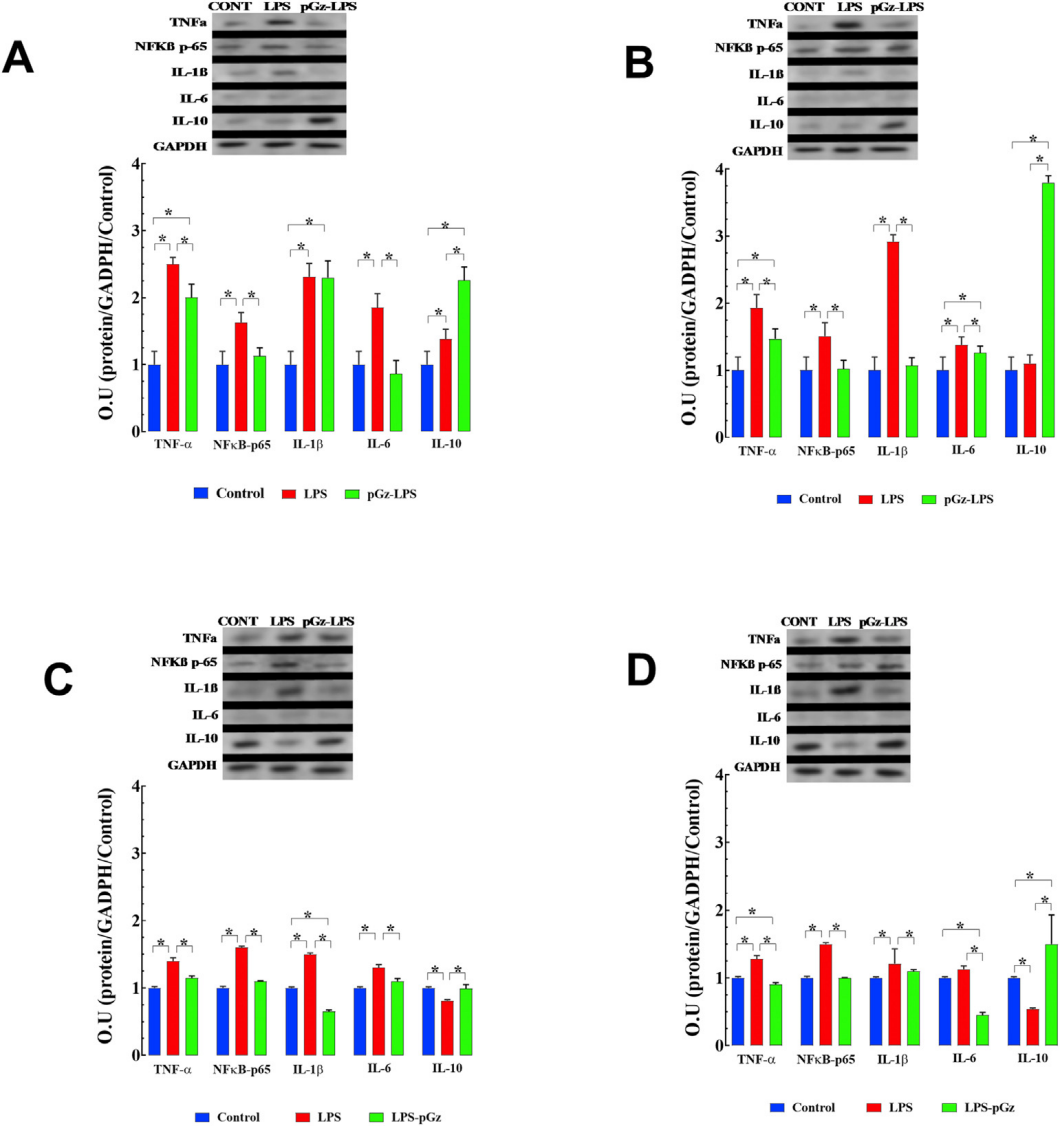
pGz attenuates LPS effects on cytokine protein expression in mice heart. LPS induced a significant increase in the expression of pro-inflammatory cytokines (TNF-α, NFkB -p65, IL-1β, and IL-6) and anti-inflammatory cytokine (IL-10). pGz pre-treatment (pGz-LPS) at 90 (A) and 360 min (B) significantly decreased expression of TNF-α, NFkB -p65, IL-6 and increases IL-10. pGz post-treatment (LPS-pGz) also significantly reduced expression of TNF-α, NFkB -p65, IL-6, and elevated IL-10 at 90 (C) and 360 min (D) after LPS. Supporting Information File contains uncropped Western Blots. Data are expressed as mean ± SD (∗p < 0.05).

Pro-inflammatory cytokines TNF-α, NFkB -p65, IL-1β, and IL-6, were markedly increased from control, 90 min after LPS by 40%,63%, 50%, and 60%, respectively. Pre-treatment with pGz reduced TNF-α, NF-ĸβ-p65, and IL-6 from LPS levels by 18%, 31%, 60% change from LPS, respectively (Figure 4, A). The anti-inflammatory cytokine IL-10 was not significantly changed by LPS but was significantly increased (22% change from LPS) by pre-treatment with pGz (Figure 4A).

Pro-inflammatory cytokines 360 min after LPS increased; TNF-α, NFkB -p65, IL-1β, and IL-6, were increased 28%, 50%, 21%, 13% change from control respectively. Pre-treatment with pGz reduced TNF-α, NFkB-p65, and IL-1β by 30%, 33%, 10%, and 60% change from LPS levels, respectively. pGz-LPS also markedly increased IL-10 by 245% change from LPS levels (Figure 4B).

At early (90 min) and late (360 min) time points after LPS, post-treatment with pGz increased p-eNOS by 142% and 100% change respectively, compared to LPS levels. iNOS upregulation in LPS-pGz by 33%and 16% change compared to LPS values at both 90 and 360 min after LPS. nNOS was increased in pGz-LPS by 38% and 46% change from LPS levels at 90 and 360 min after LPS (Figures 3C, D).

Pro-inflammatory cytokines, TNF-α, NFkB-p65, IL-1β, and IL-6 were similarly decreased at both 90 and 360 min after LPS in pGz post-treated animals. Similar to pGz-LPS, anti-inflammatory cytokine IL-10 was markedly increased in LPS-pGz, particularly 360 min after LPS (Figure 4C, D).

## 4 Discussion

The present study demonstrates that LPS induces contractile dysfunction in isolated cardiomyocytes, with intracellular [Ca^2+^] _d_ and [Na^+^] _d_ overload. LPS also increases ROS production, and increases cTnT (a marker of myocardial injury). Passive movement of the body as produced by pGz reduced these effects. The beneficial effects of pGz were mediated by NO since NOS inhibition attenuated them. Further, pGz as a pre- or post-treatment strategy significantly increased eNOS, p-eNOS, and nNOS and decreased iNOS with an associated decrease in pro-inflammatory cytokines and increase in the anti-inflammatory cytokine, IL-10.

### 4.1 Effects of pGz and NOS inhibition on LPS induced [Ca^2+^] _d,_ overload

Calcium in cardiac cells is tightly controlled both in terms of spatial and temporal distribution. Under resting conditions, [Ca^2+^]_d_ is maintained in a narrow range near 100 nM (Lopez et al., 2017., Mijares et al., 2014). To continually keep [Ca^2+^]_d_ in the nM concentration range, muscle cells use a complex dynamic equilibrium of Ca^2+^ fluxes among pumps (plasma membrane Ca^2+^ ATPase (PMCA) and sarcoplasmic reticulum Ca^2+^ ATPase (SERCA), the sarcolemma exchanger (Na^+^/Ca^2+^ exchanger), and by a voltage-independent Ca^2+^ entry. An intracellular calcium dysfunction has been previously implicated in SIC. SIC has been associated with decreased calcium currents in cardiomyocytes (Liu and Schreur, 1995), altered phosphorylation of phospholamban, decreased density of calcium L-type channels (Zhong et al., 1997), reduced responsiveness of the ryanodine receptor to calcium, and downregulation of SERCA2 (Dong et al., 2001; Martin et al., 2016; Zaky et al., 2014). pGz attenuated the effects of LPS on [Ca^2+^]_d_ and [Na^+^]_d._ and NOS inhibition with L-NAME markedly reduced the pGz effect. Thus, in large part the pGz effect on attenuation of the LPS induced cardiomyocyte [Ca^2+^]_d_ and [Na^+^]_d_ dysregulation is NO-mediated.

### 4.2 pGz reversed the LPS induced cardiomyocyte contractile dysfunction

At a functional level, SERCA2 is inhibited after endotoxemia, which is associated with a decline in ejection fraction (Hess et al., 1977; Martin et al., 2016). Prevention of downregulation of SERCA2 (by a synthetic peptide) improves impaired systolic and diastolic function in a mouse model of polymicrobial sepsis (Martin et al., 2016). Furthermore, in mice, the recovery of cardiac contractility from LPS sepsis is associated with an improvement of cardiomyocyte intracellular calcium handling (Morse et al., 2017). Our data further extends the concept of calcium dysregulation in sepsis by demonstrating a significant increase in both [Ca^2+^]_d_ and [Na^+^]_d_, with a marked decrease in peak shortening indicative of peak ventricular contractility, maximal velocities of shortening and relengthening indicatives of maximal velocities of ventricular pressure rise/fall respectively. pGz attenuated these effects via modulation of eNO and ROS levels. These findings at the cellular level concur with those found in a clinical setting (Walley, 2018). Furthermore, LPS increased a marker of myocardial injury cTnT, and pGz significantly reduced it. As expected, pGz does not modify contractile properties in ‘healthy” cardiomyocytes (Supporting Information File) (Lopez et al., 2017).

### 4.3 Effects of pGz on NOS synthases expression and restoration of LPS induced decrease in eNOS and p-eNOS

SIC is associated with a large burst of NO that is produced by iNOS. The iNOS-NO elevation provokes profound hemodynamic changes and decreases myocardial function (Cinelli et al., 2020; Spiller et al., 2019) (Ehrman et al., 2018). In contrast, NO in nanomolar quantities, produced by eNOS is cardioprotective in animal models of sepsis. Investigators have previously shown that cardiomyocyte-specific overexpression of eNOS prevents myocardial dysfunction in septic shock (Ichinose et al., 2007). Our data confirm the large increase in iNOS in sepsis and the decrease in eNOS expression and its activated form (p-eNOS). We have previously shown that a simple method of increasing sustained pulsatile shear stress with pGz increases eNOS expression and activation (Adams et al., 2005; Adams et al., 2003; Uryash et al., 2009; Wu et al., 2009; Wu et al., 2012). pGz is cardioprotective in models of ischemia-reperfusion injury (Adams et al., 2008; Martinez-Murillo et al., 2009; Uryash et al., 2012, 2015b) (Rokutanda et al., 2011) and dramatically improves survival and suppresses microvascular permeability in sepsis (Adams et al., 2019).

LPS decreases p-eNOS, whereas pGz pre- and post-treatment markedly restores both eNOS and p-eNOS. In healthy mice, we previously showed that a single 1 h session of pGz induces eNOS upregulation and phosphorylation by 20% and daily sessions for one week by 80% of control values (Uryash, Bassuk, 2015a; Wu et al., 2009). eNOS deficiency in mice increases mortality from LPS sepsis (Bougaki et al., 2010; Ichinose et al., 2007; Wang et al., 2004; Yamashita et al., 2000). Our data and others also show that LPS decreases nNOS expression (Comini et al., 2005). pGz pre- and post-treatments restores nNOS expression. nNOS is a regulator of intracellular calcium in the heart and inhibits LPS-induced TNF-α improving cardiac function in endotoxemia (Geoghegan-Morphet et al., 2007). It is important to recognize that all three NOS isoforms play a role in sepsis, but tissue location and eNOS uncoupling deserve special considerations (Duma et al., 2011; Farah et al., 2018; Gross et al., 2015; Ichinose et al., 2007; Mao et al., 2013; van de Sandt et al., 2013; Wang et al., 2004; Yamashita et al., 2000).

### 4.4 Effects of pGz on the inflammatory and anti-inflammatory cytokine response to LPS

eNOS modulates the inflammatory response, particularly that of NFkB and its crosstalk with iNOS (Perchini and Cantoni, 2006). The effects of low levels of NO (as produced by eNOS) on iNOS have been explained by others as either direct action on iNOS gene expression or via suppression of NFkB (Colasanti and Persichini, 2000; Pautz et al., 2010). LPS increases pro-inflammatory cytokines such as TNF-α, IL1β, and IL6. Additionally, NFkB-p65, a major orchestrator of the inflammatory response was also increased. These findings are not unique and are similar to other studies (Bosmann et al., 2012; Kim et al., 2014). pGz either pre- or post-treatment significantly decreased the latter both early and later after LPS. Additionally, IL-10, which is an anti-inflammatory cytokine, is decreased by LPS but markedly increased by pGz. The exact mechanisms whereby pulsatile shear stress decreases the pro-inflammatory cytokines and increases anti-inflammatory IL-10 remains to be elucidated in future studies. It is not realistic to reduce the latter findings to one single mechanism since pGz has been shown to induce a multifaceted response to cytokines and cytoprotective proteins, including antioxidant defense (Adams et al., 2018a; Uryash, Bassuk, 2015a).

### 4.5 Effects of pGz on the ROS response to LPS

Investigators have shown that LPS increases ROS production, and our data are in agreement with them (Chen et al., 2017; Steven et al., 2017). pGz reduced the LPS induced ROS surge. We previously showed that pGz treatment significantly reduced ROS production in cardiomyocytes isolated from dystrophin/utrophin double knockout (dKO) mice (Lopez et al., 2017). Furthermore, pGz in mice increases antioxidant enzymes (SOD, Catalase) expression and total antioxidant capacity (Uryash, Bassuk, 2015a). The latter may have beneficial effects on SIC and other inflammatory processes (Mantzarlis et al., 2017; Senoner and Dichtl, 2019).

### 4.6 Clinical applicability

Harnessing the endogenous production of eNO with pulsatile shear stress may have clinical applicability, particularly in the current COVID-19 pandemic caused by SARS-CoV-2 virus (Sackner and Adams, 2020). NO donor drugs have been shown to inhibit the replication of SARS-CoV-2 virus *in–vitro* (Akerstrom et al., 2005, 2009). SARS-CoV-2 targets the endothelium to produce micro- and macrovascular thrombosis, and thus COVID-19 has been proposed to be viewed as an endothelial disease (Libby and Luscher, 2020). The hallmark of endothelial disease is the suppression of eNOS with NO deficiency. Both endothelial function and improved bioavailability of NO can be accomplished with the administration of pGz (Matsumoto et al., 2008; Takase et al., 2013).

Whole body periodic acceleration motion platform (ExerRest, NIMS, Miami Fl, USA) was previously available for human use (Fujita et al., 2005; Fukuda et al., 2010; Kohler et al., 2007; Rokutanda et al., 2011), but owing to its large footprint and weight as well as providing intensive care nursing with a moving patient, a simple small device which also provides pulsatile shear stress in both seated and supine posture has been fabricated (Gentle Jogger, Sackner Wellness Products, Miami Fla, USA), which warrants study in human sepsis (Adams et al., 2018b, 2020; Sackner et al., 2019, 2020). Human trials with the latter in sepsis are needed.

### 4.7 Study limitations

This study addresses acute cytokine and selected protein response as well as cardiomyocyte response in LPS induced septic shock, longer-term response may be of interest, but given the high lethality of our model, the latter was not possible. We did not measure echocardiographic changes induced by LPS overtime in whole animal, a parameter which could provide additional insight as others have shown (Chu et al., 2019). We also did not explore the effects of pGz on the intracellular Ca^2+^ mechanisms modified by LPS (Bai et al., 2016; Luo et al., 2019; Yang et al., 2018). In a different model (swine exposed to hypoxia-ischemia cardiac arrest), we previously showed that pGz increases anti–apoptotic proteins (p-Akt, and Bcl2 and anti-apoptosis inducible factor) in the brain, however. These findings have not been confirmed in the heart (Adams, Pastuszko, 2018a) (Adams et al., 2011, 2014). Additionally, the effect of pGz on inhibition of the non-canonical inflammasome pathway was also not explored (Skirecki and Cavaillon, 2019). We also did not investigate the effects of pGz on endoplasmic reticulum stress response or unfolded protein response or the importance of the transcription factor C/EBP homologous protein 10(CHOP), which other investigators have clearly shown its importance in sepsis (Ferlito et al., 2014). Our mouse model may not directly translate to human response, as others have pointed out (Fink, 2014; Lewis et al., 2016; Remick and Ward, 2005). However, using a post-treatment strategy with pGz more closely resembles the effects of pGz after established sepsis, which is more akin to the clinical setting.

## 5 Conclusions

We conclude that pGz attenuates cardiomyocyte contractile dysfunction induced by LPS, decreases [Ca^2+^]_d,_ and [Na^+^]_d_ overload, reduces myocardial injury (cTnT), ROS production, and diminishes the inflammatory cytokine response in this model of sepsis. Human studies are needed to confirm our findings in the clinical setting.

## Declarations

### Author contribution statement

Jose Adams: Conceived and designed the experiments; Analyzed and interpreted the data; Contributed reagents, materials, analysis tools or data; Wrote the paper.

Jose R. Lopez, Arkady Uryash: Performed the experiments; Analyzed and interpreted the data; Contributed reagents, materials, analysis tools or data; Wrote the paper.

Marvin A. Sackner: Analyzed and interpreted the data; Wrote the paper.

### Funding statement

Jose Adams was supported by the Florida Heart Research Institute/Miami Heart Research.

### Data availability statement

Data will be made available on request.

### Declaration of interests statement

The authors declare no conflict of interest.

### Additional information

No additional information is available for this paper.

## Appendix A Supplementary data

Supporting Information File 3.3.2021 **Supplemental File. Protocol Schematic, pGz Platform, Behavioral Scoring Criteria and ARRIVE** Guidelines **Checklist.** This file contains a pictorial schematic of the two protocols used in the study, a schematic of the platform to impart whole body periodic acceleration (pGz) in animal models along with detailed description on the characteristics of the motion platform. The document also contains the expanded Behavioral Scoring Criteria in Mice, amended to include stool quality criteria, a maximal worse score is 32. Information on euthanasia methods and animal attrition for all experiments. A graph showing the effects of pGz and Nitric Oxide Inhibition using L-NAME. on normal cardiomyocytes contractile function. The uncropped images of blots from Figures3 and 4, along with a description of the methodology. A table also contains ARRIVE Guideline Checklist for animal research reporting for in vivo experiments. Supporting Information File 3.3.2021
