# Supplementary material for: Whole body periodic acceleration (pGz) improves endotoxin induced cardiomyocyte contractile dysfunction and attenuates the inflammatory response in mice
Source: Heliyon. 2021 Mar 12;7(3):e06444. doi: 10.1016/j.heliyon.2021.e06444 (PMC7970274; doi:10.1016/j.heliyon.2021.e06444)

Supporting Information

**Protocol Schematics**

Graphical representation of the two protocols used in the current study. 1-***LPS and pGz Cellular Data***. - Details of each groups are found in the manuscript. The purpose of this protocol was to test the pretreatment effects of pGz on cardiomyocyte contractile dysfunction induced by LPS. This protocol used pGz as a pre-treatment strategy for 3 days followed by LPS (10 mg/kg). Additionally, one group received 7 days of the NO inhibitor L-NAME along with pGz. Six hours after LPS, animals were euthanized and measurements of Peak Shortening, +dL/dt, -dl/dt, Intracellular calcium and sodium, ROS production and plasma measurements of cTnT, were performed as detailed in the manuscript. Tissue harvesting occurred 360 min after LPS 2- ***Protein Expression and Cytokines*,** we tested effects of pGz on NOS isoforms and cytokine protein expression in mice hearts at 90 and 360 min after a lethal dose of LPS (40mg/kg). Four groups were studied; a) Control (no LPS or pGz), animals received saline buffer. b) LPS(animals received only LPS) , c) pGz-LPS( animals were treated with 1 hr daily for 3 days with pGz) followed by LPS, and d) LPs-pGz ( after the LPS dose animals were treated with pGz). In this group pGz was started for 1hr immediately after the LPS dose. Tissue was harvested 90 and 360 min after buffer or LPS . In the LPS-pGz group tissue harvesting occurred 30 min after completion of pGz. See manuscript for more details.

The pGz platform is composed of a linear motor, driven by an amplifier (controller) which generates a sinusoidal output at varying frequencies and amplitudes. The shaft of the linear motor articulates a platform on slides, allowing free range of motion in the headward to tail axis(z) of the animal. Animals are placed on the platform restrained via an animal holder (Kent Scientific), and whole body periodic acceleration (pGz) is imparted for 30 min. Details of the frequency and acceleration forces used are in the manuscript.

The axis of motion of the platform is always in the horizontal Z- axis (head to tail), thus the term coined by our laboratory of periodic gravitational forces in the Z plane (pGz), also known as whole body periodic acceleration (WBPA). The table below reports the commonly used descriptors as recommended by the International Society of Musculoskeletal and Neuronal Interactions for reporting whole body vibration interventions [[1](#_ENREF_1)] for the present study.

|  |  |  |  | **AXIS** | | |
| --- | --- | --- | --- | --- | --- | --- |
|  |  |  |  | **X** | **Y** | **Z^*^** |
| **Parameter** | **Unit** | **Symbol** |  | **8 Hz (Z)** | **8 Hz (Z)** | **8 Hz (Z)** |
| **Period duration** | s | T |  | ***0.125*** | ***0.125*** | ***0.125*** |
| **Frequency** | Hz, s^2-1 | f |  | ***8*** | ***8*** | ***8*** |
| **Peak to Peak Displacement** | mm | D |  | ***0.31*** | ***0.004*** | ***2.4*** |
| **Amplitude** | mm | A |  | ***0.16*** | ***0.002*** | ***1.2*** |
| **Peak Acceleration** | ms^-2 | a(Peak) |  | ***0.39*** | ***0.005*** | ***3*** |
| **Peak Acceleration** | g | a(Peak) |  | ***0.04*** | ***0.0005*** | ***0.31*** |
| **Root Mean Squared Acceleration (RMSA)** | ms^-2 | a(RMS) |  | ***0.74*** | ***0.028*** | ***2.49*** |

*Note that the pGz imparts Z-Axis Acceleration, with minimal to negligible X or Y axis acceleration.

# Humane Endpoint Criteria, Euthanasia and Animal Attrition

Humane endpoints were pre-defined in the protocol using the modified behavioral criteria as below. Behavioral scoring was performed in all animals for the initial 48 hrs. after LPS injection. The Behavioral Scoring criteria utilized has been described by Shrum et al [[2](#_ENREF_2)]. The Behavioral Scoring was amended to include stool quality as additional criteria, with a maximum score of 32. Animals were humanely euthanized within 15 min once a score of 28 was reached. Behavioral Scoring was performed every 30 min after LPS for the first 2 hrs, thereafter every 1hr for until 6 hrs.

# Behavioral Scoring Criteria

The below criteria was modified from Shrum et al to include stool quality. The maximum score is 32.[[2](#_ENREF_2)]

| **Variable** | **Score and description** |
| --- | --- |
| Appearance | 0- Coat is smooth |
|  | 1- Patches of hair piloerected |
|  | 2- Majority of back is piloerected |
|  | 3- Piloerection may or may not be present, mouse appears “puffy” |
|  | 4- Piloerection may or may not be present, mouse appears emaciated |
| Level of consciousness | 0- Mouse is active |
|  | 1- Mouse is active but avoids standing upright |
|  | 2- Mouse activity is noticeably slowed. The mouse is still ambulant. |
|  | 3- Activity is impaired. Mouse only moves when provoked, movements have a tremor |
|  | 4- Activity severely impaired. Mouse remains stationary when provoked, with possible tremor |
| Activity | 0- Normal amount of activity. Mouse is any of: eating, drinking, climbing, running, fighting |
|  | 1- Slightly suppressed activity. Mouse is moving around bottom of cage |
|  | 2- Suppressed activity. Mouse is stationary with occasional investigative movements |
|  | 3- No activity. Mouse is stationary |
|  | 4- No activity. Mouse experiencing tremors, particularly in the hind legs |
| Response to stimulus | 0- Mouse responds immediately to auditory stimulus or touch |
|  | 1- Slow or no response to auditory stimulus; strong response to touch (moves to escape) |
|  | 2- No response to auditory stimulus; moderate response to touch (moves a few steps) |
|  | 3- No response to auditory stimulus; mild response to touch (no locomotion) |
|  | 4- No response to auditory stimulus. Little or no response to touch. Cannot right itself if pushed over |
| Eyes | 0- Open |
|  | 1- Eyes not fully open, possibly with secretions |
|  | 2- Eyes at least half closed, possibly with secretions |
|  | 3- Eyes half closed or more, possibly with secretions |
|  | 4- Eyes closed or milky |
| Respiration rate | 0- Normal, rapid mouse respiration |
|  | 1- Slightly decreased respiration (rate not quantifiable by eye) |
|  | 2- Moderately reduced respiration (rate at the upper range of quantifying by eye) |
|  | 3- Severely reduced respiration (rate easily countable by eye, 0.5 s between breaths) |
|  | 4- Extremely reduced respiration (>1 s between breaths) |
| Respiration quality | 0- Normal |
|  | 1- Brief periods of labored breathing |
|  | 2- Labored, no gasping |
|  | 3- Labored with intermittent gasps |
|  | 4- Gasping |
| Stool | 0- Normal |
|  | 1- Frequent or a lot |
|  | 2- Loose |
|  | 3- Diarrhea |
|  | 4- No stool |
| TOTAL | Score |
|  | **MAX WORSE SCORE 32** |

# Euthanasia

After completion of each of the experimental protocols or after reaching the humane endpoint of behavioral score of 28 , animals were euthanized by a dose of Ketamine 90mg/kg and Xylazine 25mg/kg, followed by pentobarbital 100mg/kg IP, until absence of corneal and pedal reflex, and no electrical activity on ECG, and decapitation via guillotine, a method approved by the American Veterinary Medical Association Guidelines on Euthanasia. Perfusion and or organ harvesting was performed as per protocol [[3-5](#_ENREF_3)]

# Animal Attrition

***LPS and pGz Cellular Data***

|  |  | ***Required Euthanasia*** |
| --- | --- | --- |
| ***Animal Group*** | ***N*** | ***Prior Completion of Protocol*** |
| Control | 5 | 0 |
| pGz | 5 | 0 |
| LPS | 5 | 0 |
| pGz-LPS | 5 | 0 |
| L-NAME- pGz-LPS | 5 | 0 |
| ***TOTALS*** | ***N=25*** |  |

***Protein Expression and Cytokines***

| Animal Group | Euthanized @90 min (n) | Euthanized @ 360min (n) | Required Humane Euthanasia Prior to Endpoint | Totals (N) |
| --- | --- | --- | --- | --- |
| pGz-LPS | 8 | 8 | 0 | 16 |
| LPS-pGz | 8 | 8 | 0 | 16 |
| LPS | 8 | 8 | 0 | 16 |
| Sham | 8 | 8 | 0 | 16 |
|  |  |  | ***TOTALS*** | ***64*** |

**Statistical Considerations and Sample Size Calculation**

*Statistical Consideration for Measurements of [Ca^2+^]_d,_ and [Na^+^]_d_*

All values are expressed as means± SEM, with n_cells_ representing the number of cardiomyocytes in which successful measurements were carried out and N_mice_ representing the number of mice used to isolate the cardiomyocytes. The number of measurements (n) reported are those measurements that meet the criteria established i) an abrupt drop to a steady level of Vm equal to or more negative than -80 mV,ii) a stable recording of both Vm and Ca^2+^or Na^+^ potential for more than 1 min and iii) an abrupt return to baseline on the exit of the microelectrode from the cardiomyocytes. In addition, measurements were rejected if the initial and final calibration curves did not agree within 3 mV. The reported values are based on cardiomyocytes isolated from the 5 mice used in each experimental group.

*Sample Size Calculation*

Our past experience with pGz has shown that we can minimally anticipate a 20% change in protein of interest, calcium measurements, ROS or cTnT. Based upon the latter with a 10% standard deviation with an α = 0.05 and power 0.80, Beta 0. 2 the minimum sample size is N_mice_=4 per group. We have used N=8 animals for each group and each time point

# The effects of pGz and L-NAME on isolated cardiomyocyte contractility

We have previously shown that pGz performed in normal mice does not change cardiomyocyte contractile properties or [Ca^+2^]_d_ . In order to confirm our previous findings and to ascertain the degree of NO inhibition produced by the oral dose of the non-selective nitric oxide inhibitor (L-NAME) we performed a separate group pf experiments. Mice C57BL6J ( The Jackson Laboratory) n=20 or either sex 3 months of age of weight between 20-25gms were randomized ( via computer generated codes) to 4 groups: a) Three consecutive days of pGz ( 1 hr per day) as previously described [pGz], b) Control ( no pGz but were placed in the same animal holder as used for pGz daily for 1 hr for 3 days) [Control] , c) L-NAME , (1.5mg/ml) was given in their drinking water for 7 days [L-NAME] (in this group we tested the short term effects of L-NAME) , d) Combined L-NAME and pGz, in this group L-NAME was given in the drinking water for 7 days and in the last 3 days pGz was administered daily for 3 days.[L-NAME-pGz]. Tissue was harvested , cells processed, and contractile measurements performed as described in the manuscript. We confirm our previous findings that pGz does not modify contractile properties of a normal cardiomyocyte [[6](#_ENREF_6)]. We also confirmed that the current dose of L-NAME used in this protocol reduced NO production such that pGz when given simultaneously was unable to improve contractile response.


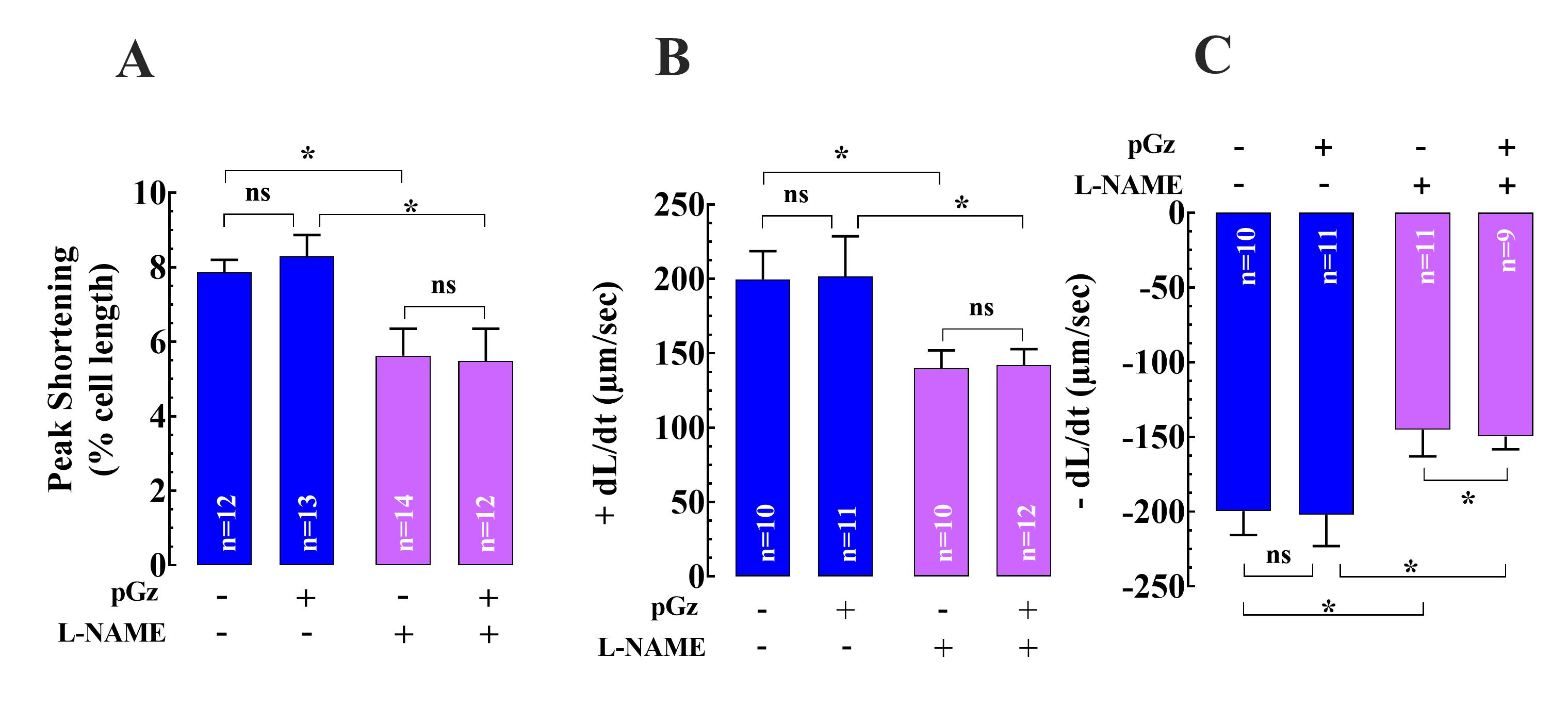
The effects of pGz and L-NAME on cardiomyocyte contractile function were tested in quiescent normal cardiomyocytes (not exposed to LPS). As expected, pGz had no effect on the measured contractile properties. The non-selective NOS inhibitor L-NAME significantly reduced peak shortening **(A**), +dL/dt **(B)**, and –dL/dt **(C)**. Addition of pGz to L-NAME was unable to abrogate the reduction of contractile properties induced by L-NAME, confirming that the dose of L-NAME was appropriately inhibitory in this model.

# Western Blot Analysis of LPS exposed cardiomyocytes, pre-treated (pGz-LPS) and post treated (LPS-pGz) with pGz.

Western Blot Methodology:

Hearts from the high dose LPS were harvested at 90 and 360 min after LPS infusion and processed for protein analysis using western blot techniques previously reported. [[7](#_ENREF_7),[8](#_ENREF_8)]. These periods were chosen in order to have two specific periods with adequate survival of animals in both treated and control groups based on survival data [[8](#_ENREF_8)]. Briefly, homogenized mouse hearts were processed using a one-step protein extraction kit (Millipore Corporation, Billerica, MA). Then total protein concentrations were measured by the BCA Protein Assay (Thermo Fisher Scientific, Waltham, MA, USA) on a SpectraMax Plate Reader (Molecular Devices, Sunnyvale, CA, USA). Individual proteins of interest were then analyzed by western blot. Equal amounts of total protein were separated on 4–12% NuPAGE Novex Bis-Tris SDS-PAGE Gels (Life Technologies, Carlsbad, CA, USA) and transferred to nitrocellulose membrane (Bio-Rad, Hercules, CA, USA). The transfer membrane was treated with a blocking agent (GE Bio-Sciences, Piscataway, NJ, USA) and probed with primary, fluorescein-linked secondary antibodies as well as anti-fluorescein alkaline phosphatase conjugate.

The following primary antibodies were used: eNOS (5589, 1:2000), p-eNOS (184154, 1:2000 Ser 1177), nNOS (5586, 1:2000), iNOS (15323, 1:2000), TNFα (6671, 1:5000), IL-1β (9787, 1:2000), IL-6 (9324, 1:2000), IL-10 (9969, 1:2000), NFkB-p65 (32536, 1:1000), GAPDH (9545, 1:10000) (Abcam, Cambridge, MA, USA). Protein signals were visualized using Enhanced Chemifluorescence kit (ECF) and Storm 860 Imaging System (GE Bio-Sciences, Piscataway, NJ, USA). The Storm 860 Imaging System exhibits a linear response to two fluorescent signal intensities. Two distinct absorbance frequencies were used. Combination of two different emission frequencies fluorescent antibodies, blot stripping and molecular weight range specific incubation, allows us to obtain multiple targets imaging from the same blot run. Additionally, we cut the membrane (horizontally) for signal background leveling and image analysis. Size is determined based on protein marker lines. Protein levels were quantified using MYImageAnalysis software (Thermo Fisher Scientific, Waltham, MA, USA). Optical units were first standardized to GAPDH protein loading control and then referenced to control levels of individual proteins. In this supplement we present the uncropped blots.

The molecular weight of the proteins under study are:

|  | kDa |
| --- | --- |
| nNOS | 190 |
| p-eNOS | 150 |
| eNOS | 140 |
| iNOS | 130 |
| NFKb-p65 | 65 |
| GAPDH | 40 |
| IL-1b | 31 |
| TNF | 28 |
| IL-6 | 24 |
| IL-10 | 20 |

**Legend for Western Blot Panels:** *The following are the uncropped blots which contain the proteins probed and reported in the manuscript for Figures 3 and 4. The individual blots were labeled for clarity. Each panel (A thru H) contain 5 blots. Each blot contains proteins of interest from tissue harvested at 90 min (left side of blot) and 360 min (right side of blot). The molecular marker is labeled as M. Each lane of the blot contains protein samples which are labeled as follows; M (Molecular Weight Marker, in kilo Dalton (kD), lanes 1, 4,7,10 (CONT=Control) lanes 2,5,8,11 (LPS=Lipopolysaccharide), lanes 3, 6 (LPS-pGz animals post treated with pGz) and lanes 9,12 (pGz-LPS animals pretreated with pGz). Panels A, B, C, D contains tissue of animals post treated with pGz (lanes 3,6) and Panels E,F,G,H contains tissue of animals pretreated with pGz (lanes 9,12). The green box in each of the panels denotes the blot which is utilized as a representative example in Figures 3, and 4 in the manuscript.*

**
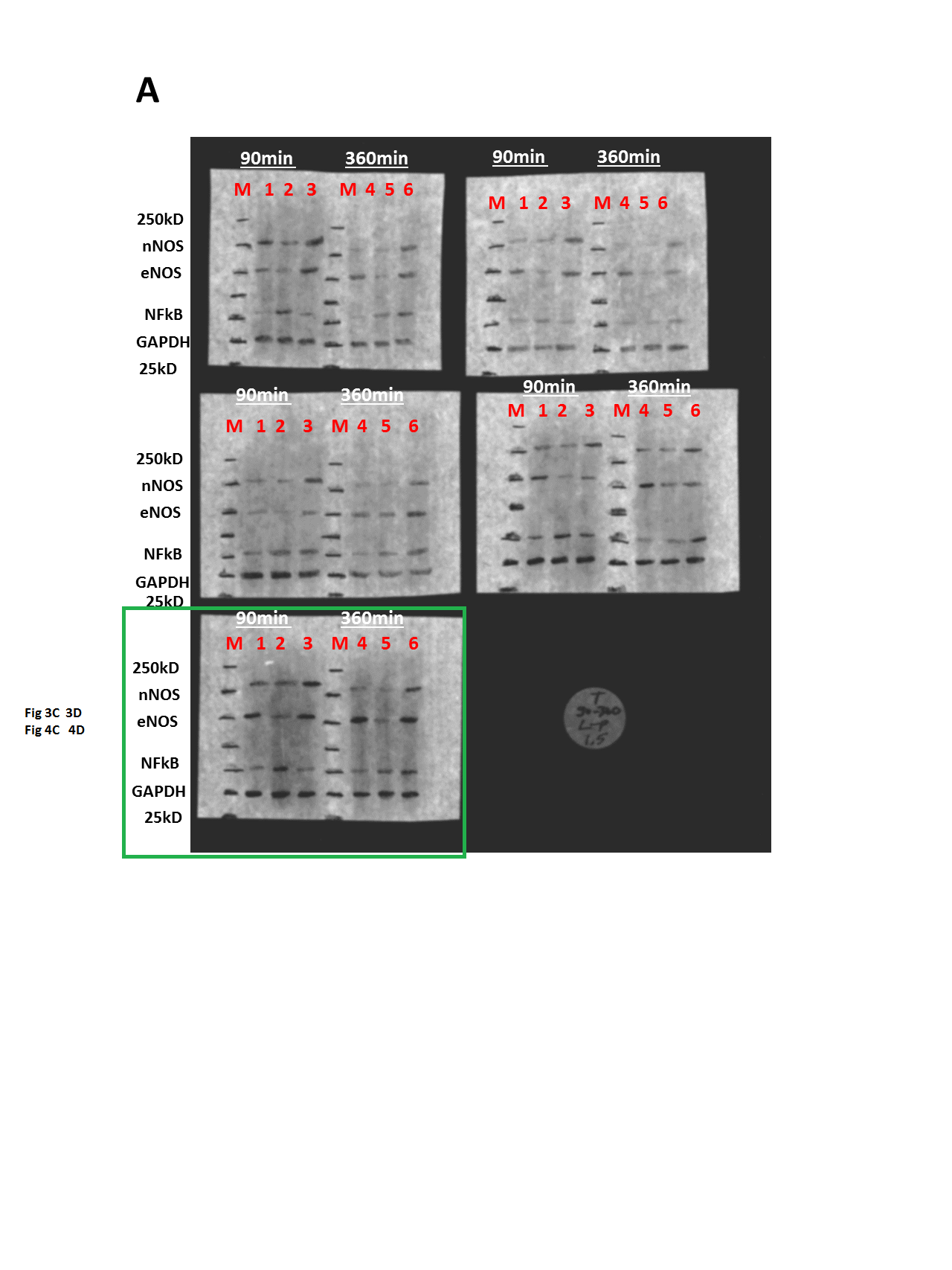
**

**
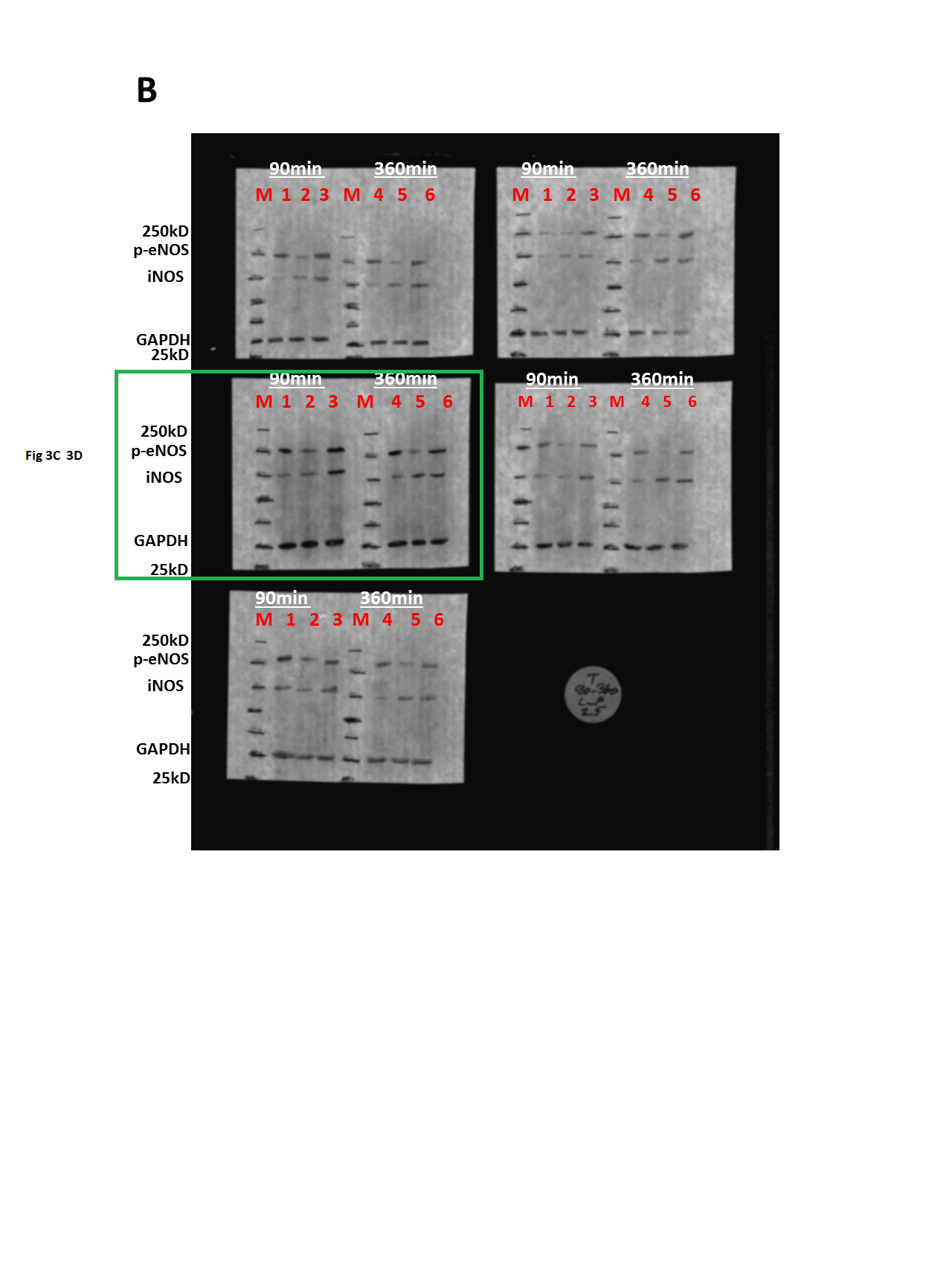
**

**
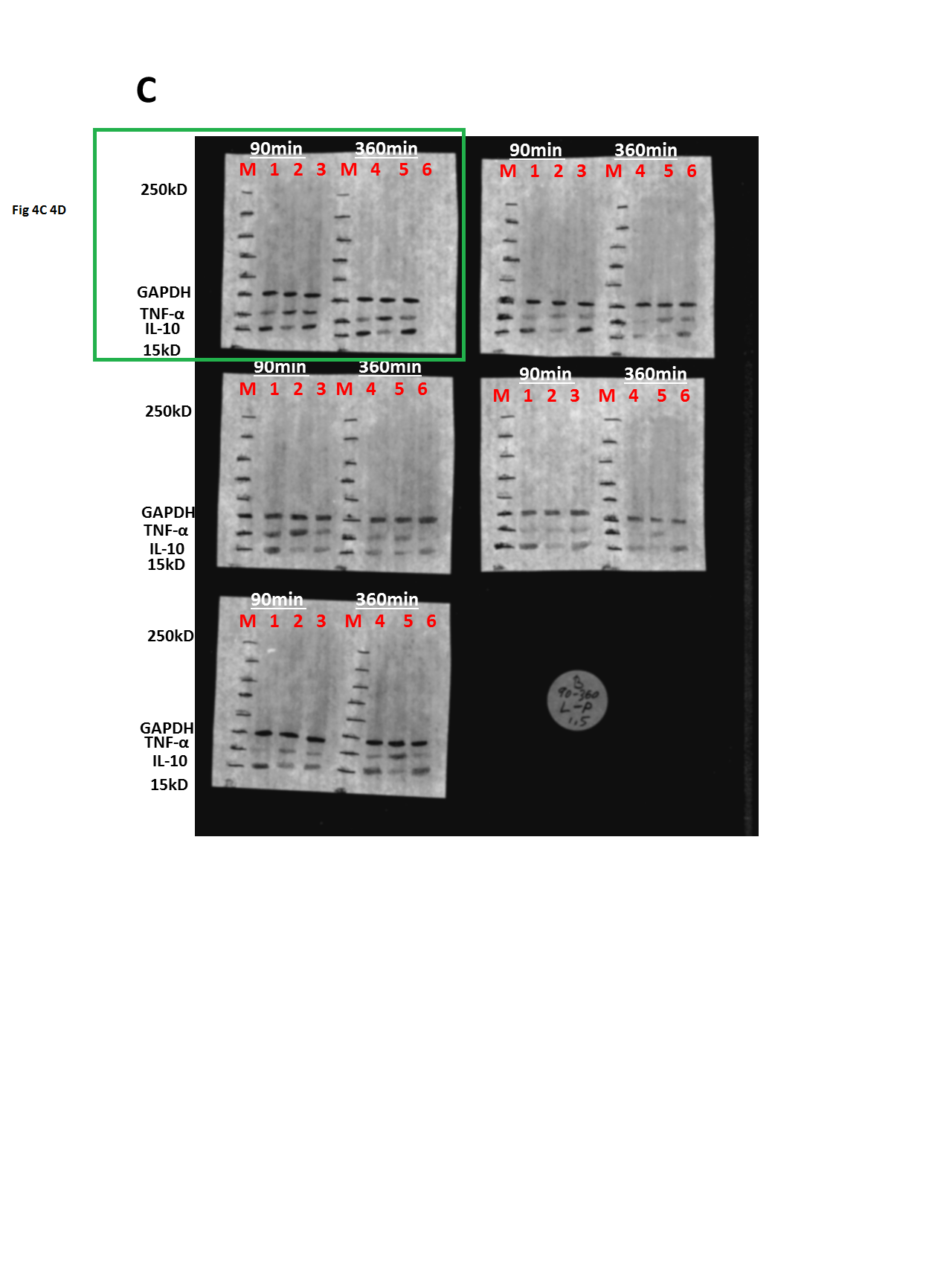
**

**
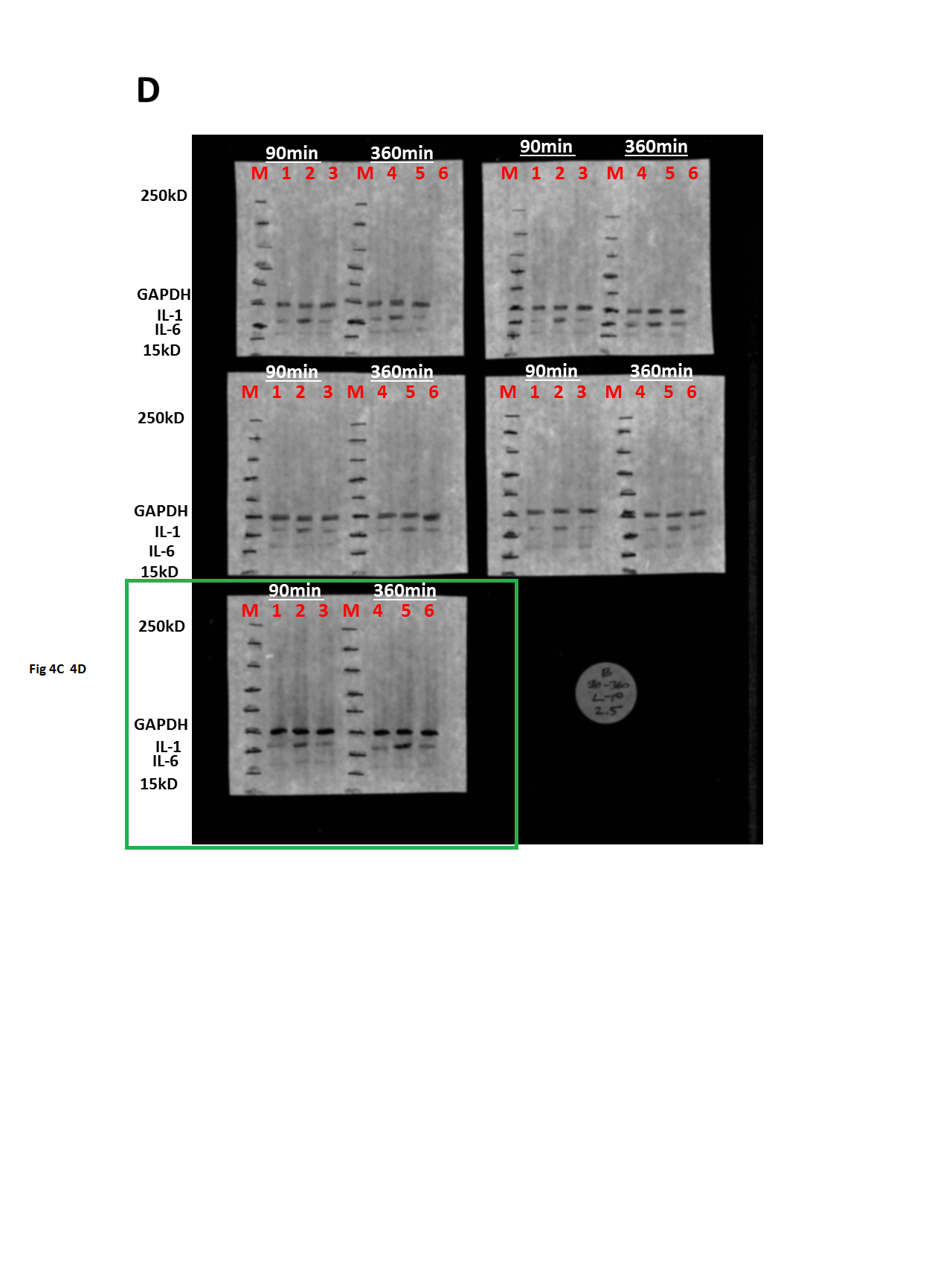
**

**
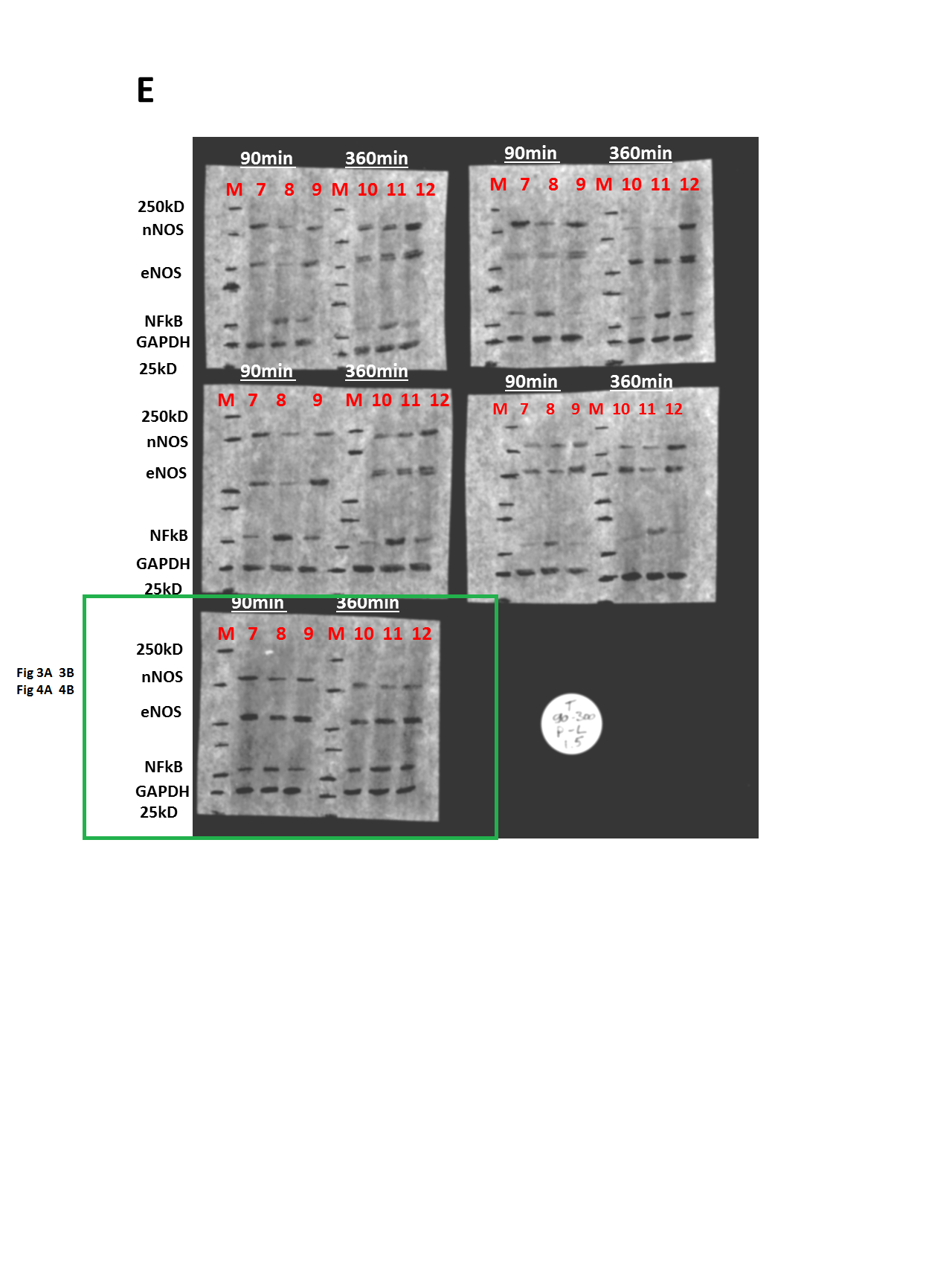
**

**
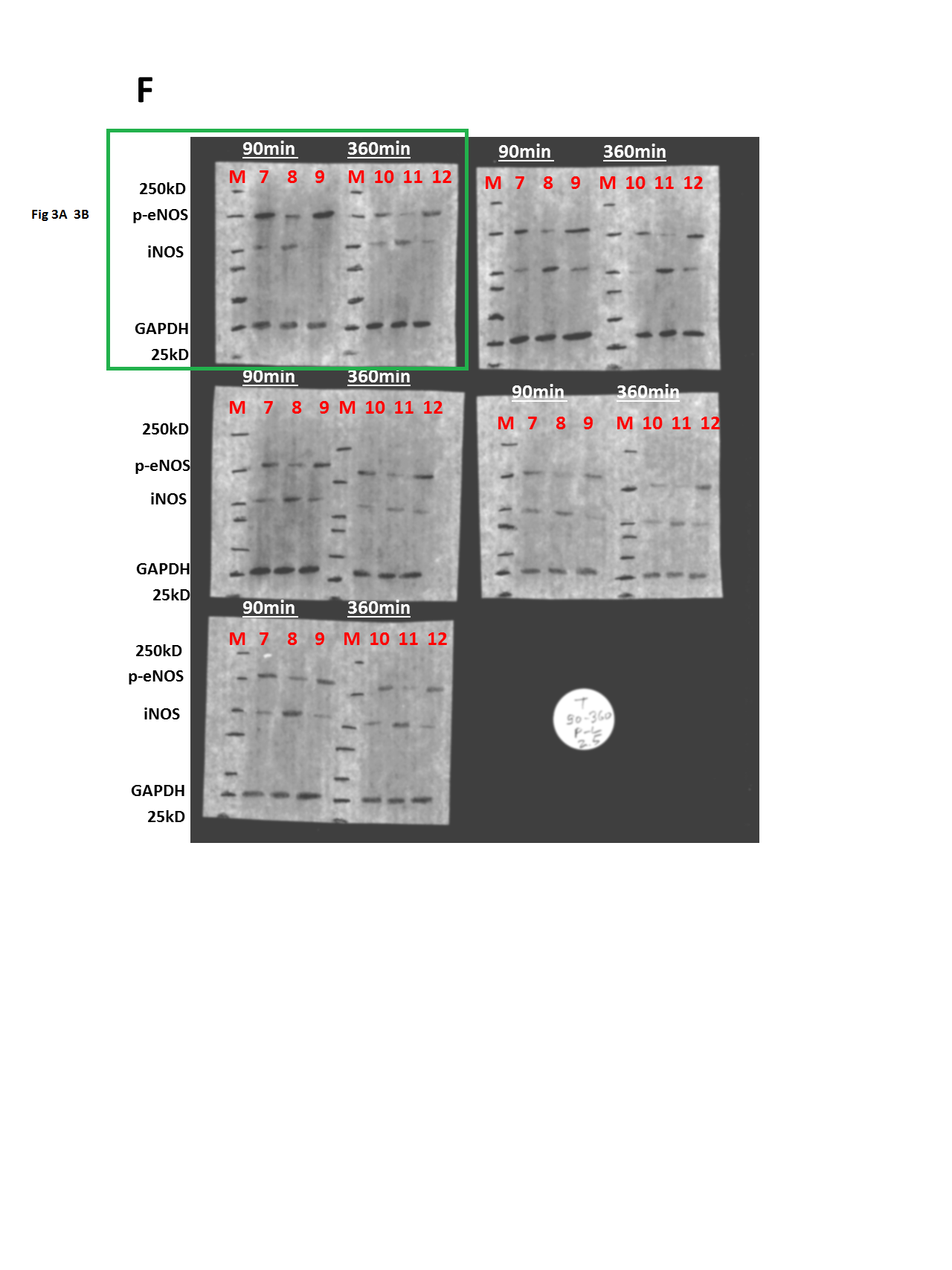
**

**
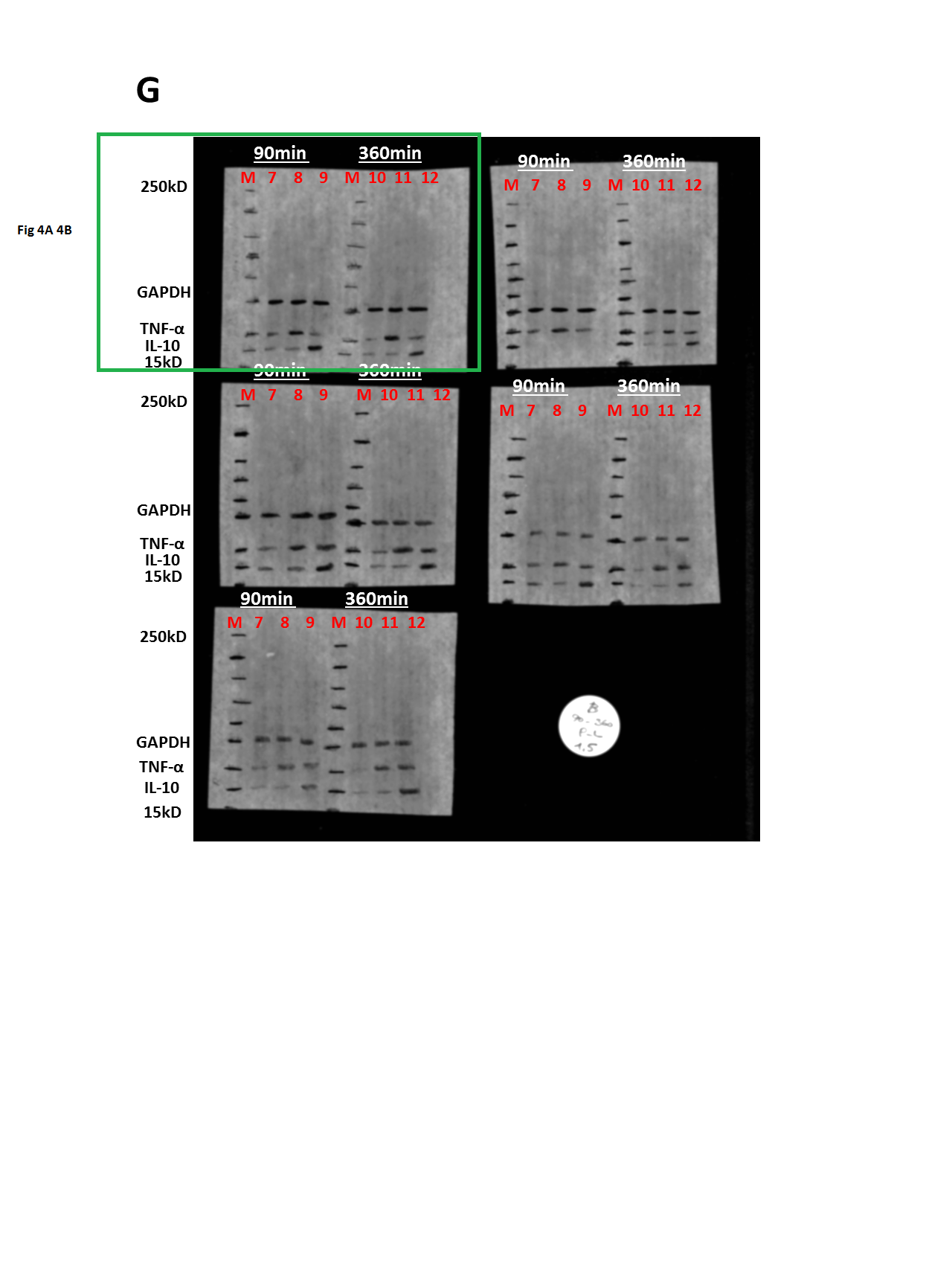
**

**
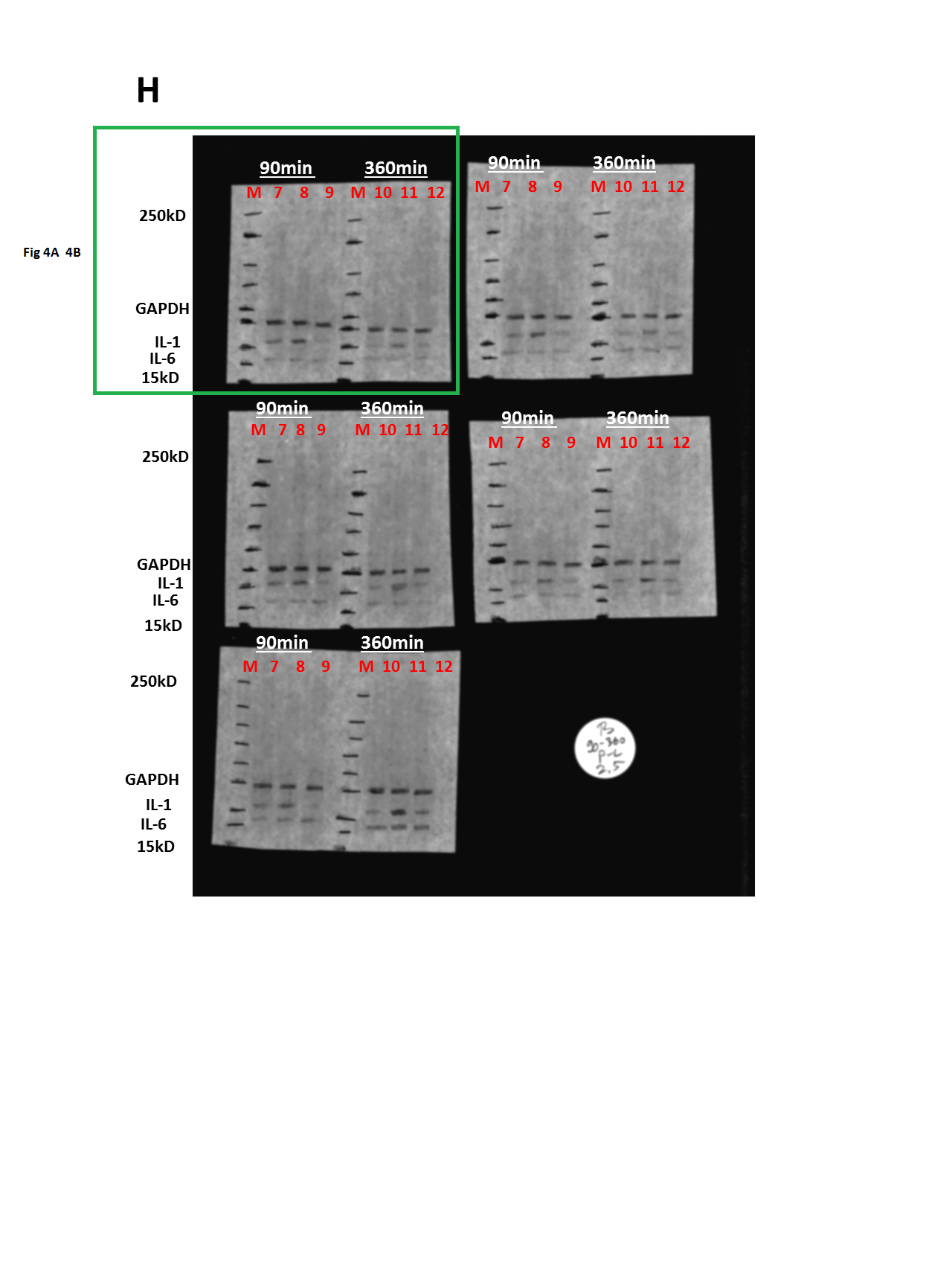
**

**REFERENCES**

1. Rauch F, Sievanen H, Boonen S, Cardinale M, Degens H, Felsenberg D, Roth J, Schoenau E, Verschueren S, Rittweger J, International Society of M, Neuronal I (2010) Reporting whole-body vibration intervention studies: recommendations of the International Society of Musculoskeletal and Neuronal Interactions. J Musculoskelet Neuronal Interact 10 (3):193-198

2. Shrum B, Anantha RV, Xu SX, Donnelly M, Haeryfar SM, McCormick JK, Mele T (2014) A robust scoring system to evaluate sepsis severity in an animal model. BMC research notes 7:233. doi:10.1186/1756-0500-7-233

3. Nemzek JA, Xiao HY, Minard AE, Bolgos GL, Remick DG (2004) Humane endpoints in shock research. Shock 21 (1):17-25. doi:10.1097/01.shk.0000101667.49265.fd

4. Toth LA (2000) Defining the Moribund Condition as an Experimental Endpoint for Animal Research. ILAR journal / National Research Council, Institute of Laboratory Animal Resources 41 (2):72-79

5. Morton DB (2000) A systematic approach for establishing humane endpoints. ILAR journal / National Research Council, Institute of Laboratory Animal Resources 41 (2):80-86

6. Lopez JR, Kolster J, Zhang R, Adams J (2017) Increased constitutive nitric oxide production by whole body periodic acceleration ameliorates alterations in cardiomyocytes associated with utrophin/dystrophin deficiency. Journal of molecular and cellular cardiology 108:149-157. doi:10.1016/j.yjmcc.2017.06.004

7. Uryash A, Bassuk J, Kurlansky P, Altamirano F, Lopez JR, Adams JA (2015) Antioxidant Properties of Whole Body Periodic Acceleration (pGz). PLoS One 10 (7):e0131392. doi:10.1371/journal.pone.0131392

8. Adams JA, Uryash A, Lopez JR, Sackner MA (2019) Whole body periodic acceleration improves survival and microvascular leak in a murine endotoxin model. PLoS One 14 (1):e0208681. doi:10.1371/journal.pone.0208681

The ARRIVE Guidelines Checklist

Animal Research: Reporting In Vivo Experiments

Carol Kilkenny^1^, William J Browne^2^, Innes C Cuthill^3^, Michael Emerson^4^ and Douglas G Altman^5^

*^1^The National Centre for the Replacement, Refinement and Reduction of Animals in Research, London, UK, ^2^School of Veterinary Science, University of Bristol, Bristol, UK, ^3^School of Biological Sciences, University of Bristol, Bristol, UK, ^4^National Heart and Lung Institute, Imperial College London, UK, ^5^Centre for Statistics in Medicine, University of Oxford, Oxford, UK.*

|  | | ITEM | RECOMMENDATION | Section/ Paragraph |
| --- | --- | --- | --- | --- |
| 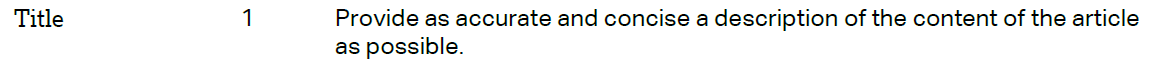 | | | 1 |  |
| 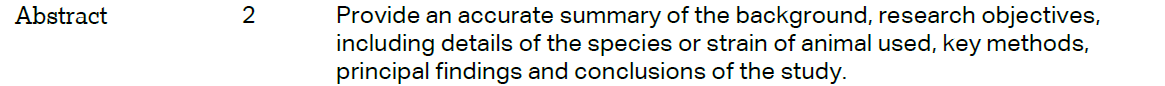 | | | 2-3 |  |
| INTRODUCTION | | |  |  |
| 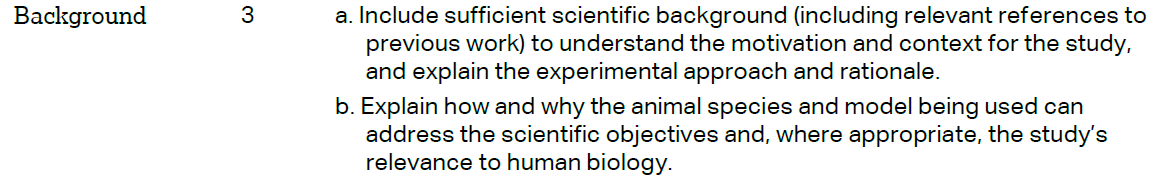 | | | 3 |  |
| 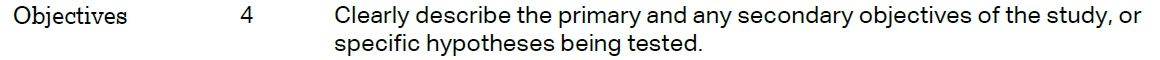 | | | 4-5 |  |
| METHODS | | |  |  |
| 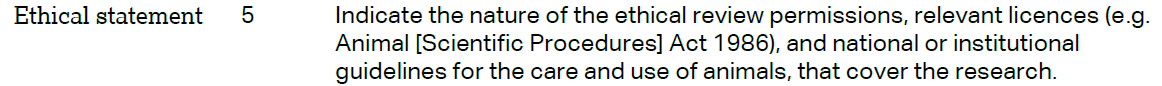 | | | 4 |  |
| 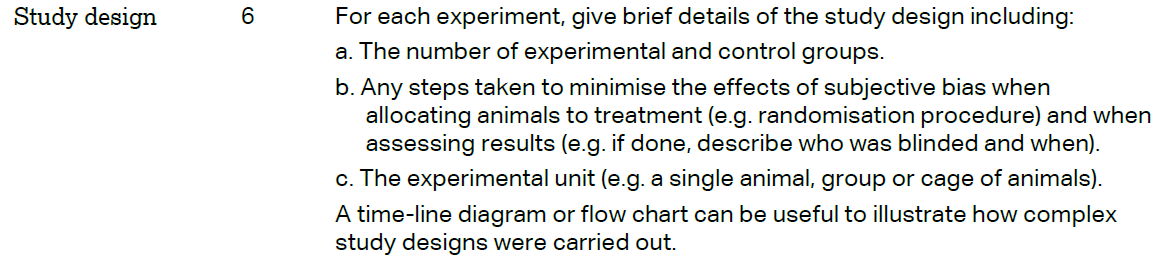 | | | 5 and Supplemental File |  |
| 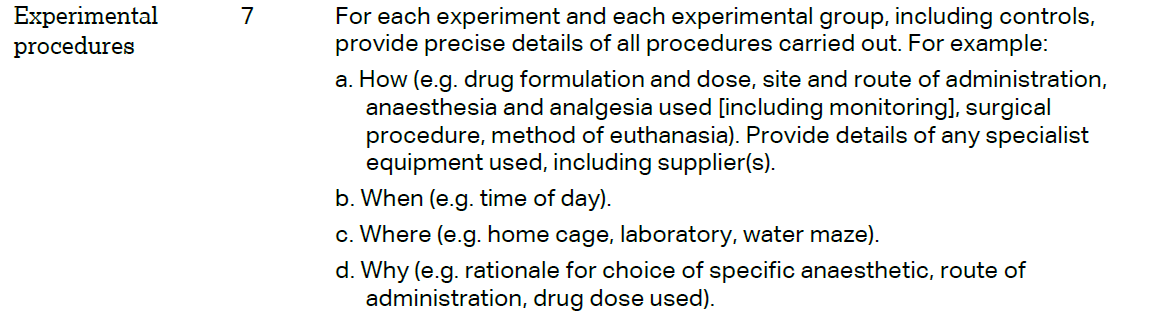 | | | 6-9 and Supplemental File |  |
| 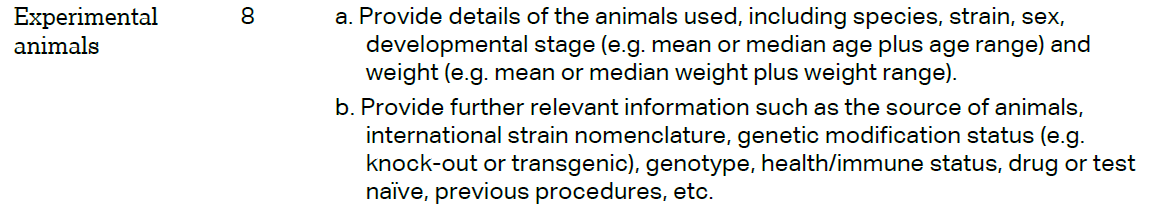 | | | 5 and Supplemental File |  |

The ARRIVE guidelines. Originally published in *PLoS Biology*, June 2010^1^

| 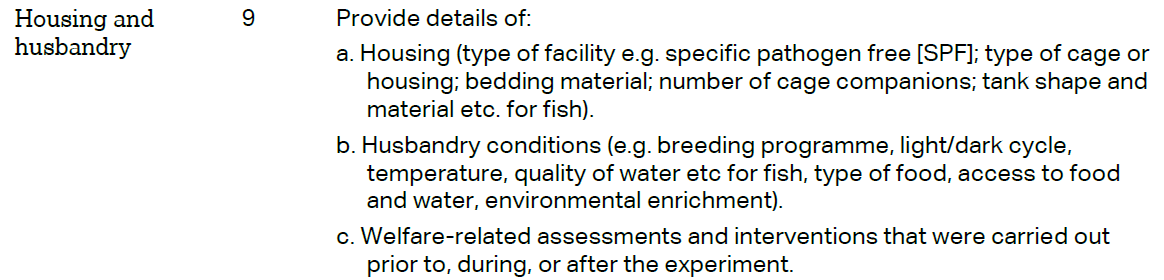 | 5-6 and supplemental file | |
| --- | --- | --- |
| 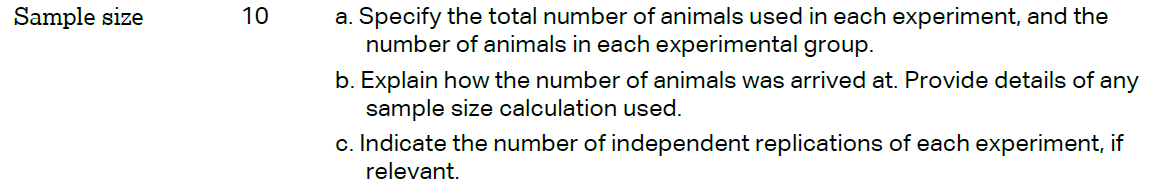 | 5-6  Supplemental File | |
| 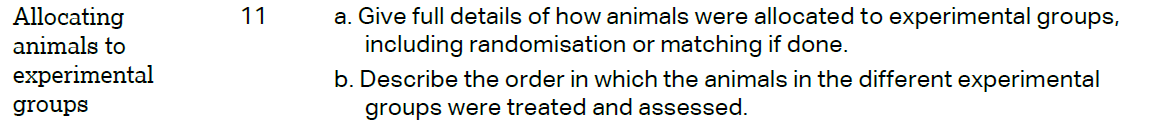 | 5-6 and Supplemental file | |
| 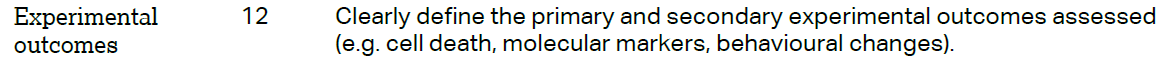 | 6-9 and Supplemental File | |
| 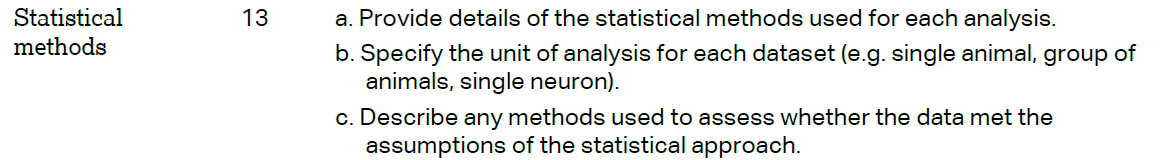 | 9 and Supplemental File | |
| RESULTS |  | |
| 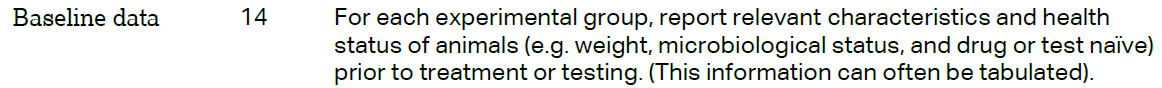 | 10-12 | |
| 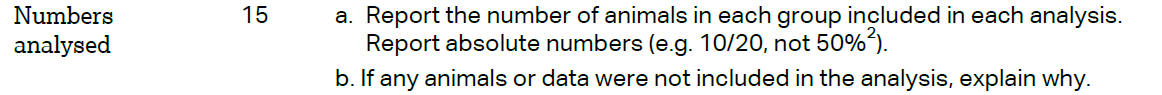 | 10-13 and Supplemental file and Figures | |
| 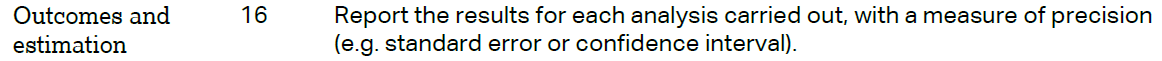 | 10-13 Supplemental File and Figures | |
| 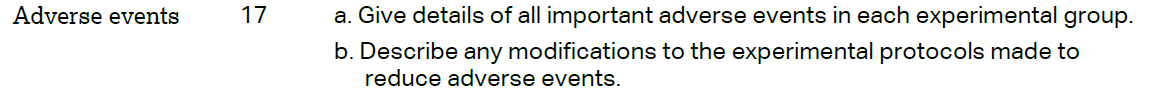 | N/A | |
| DISCUSSION |  | |
| 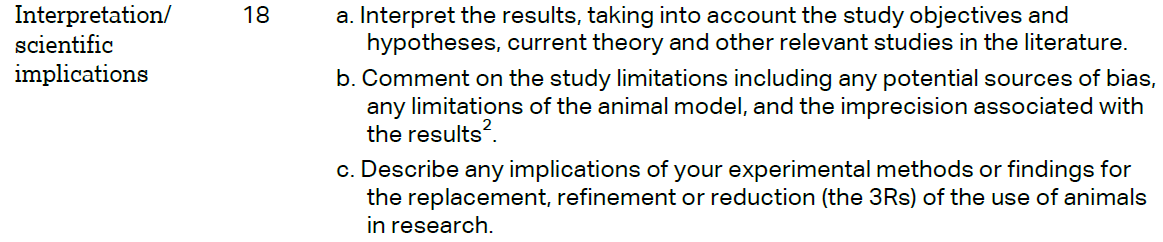 | 13-18 | |
| 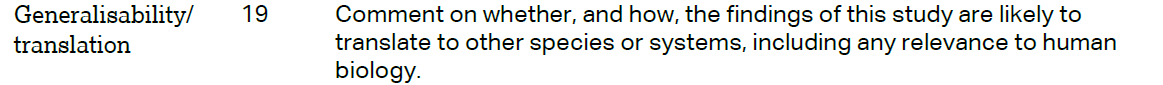 | 16-17 | |
| 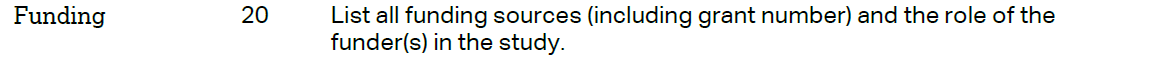 | | 22 |


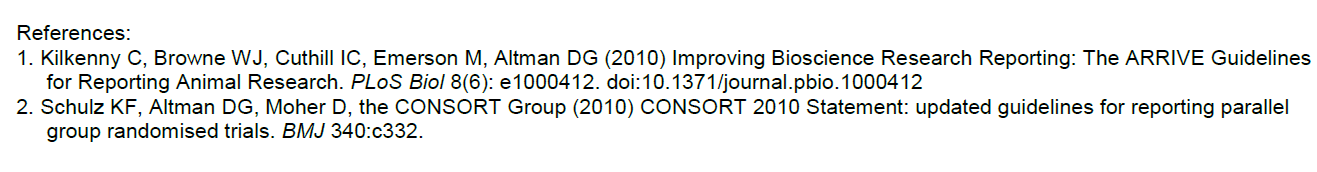

Supplement: Supporting Information File 3.3.2021 — Supplemental File. Protocol Schematic, pGz Platform, Behavioral Scoring Criteria and ARRIVE Guidelines Checklist. This file contains a pictorial schematic of the two protocols used in the study, a schematic of the platform to impart whole body periodic acceleration (pGz) in animal models along with detailed description on the characteristics of the motion platform. The document also contains the expanded Behavioral Scoring Criteria in Mice, amended to include stool quality criteria, a maximal worse score is 32. Information on euthanasia methods and animal attrition for all experiments. A graph showing the effects of pGz and Nitric Oxide Inhibition using L-NAME. on normal cardiomyocytes contractile function. The uncropped images of blots from Figures3 and 4, along with a description of the methodology. A table also contains ARRIVE Guideline Checklist for animal research reporting for in vivo experiments. [file mmc1.docx]
